# PBA-enriched glycated/ glycosylated synovial fluid proteomic signatures associated with metabolic dysregulation and cartilage degeneration in osteoarthritis with type 2 diabetes

**DOI:** 10.1186/s12967-026-07984-8

**Published:** 2026-03-25

**Authors:** Ayushi Sharma, Uttam Chand Saini, Sanjay Kumar Bhadada, Sadhna Sharma, Jyotdeep Kaur

**Affiliations:** 1 Department of Biochemistry, Postgraduate Institute of Medical Education and Research (PGIMER), Chandigarh, 160012 India; 2 Department of Orthopedics, Postgraduate Institute of Medical Education and Research (PGIMER), Chandigarh, 160012 India; 3 Department of Endocrinology, Postgraduate Institute of Medical Education and Research (PGIMER), Chandigarh, 160012 India; 4 Department of Biochemistry, Maharishi Markandeshwar Institute of Medical Sciences and Research, Mullana, Haryana 133207 India

**Keywords:** Glycation, Glycosylation, Proteomics, Mass spectrometry, Osteoarthritis, Type 2 diabetes mellitus, ECM remodeling, Inflammation

## Abstract

**Background:**

Osteoarthritis (OA) frequently co-exists with type 2 diabetes mellitus (T2DM) owing to shared risk factors, including age and obesity, and disease severity is often greater in individuals with both conditions (OADM). However, molecular alterations associated with this co-morbidity remain incompletely understood. Here, we applied a proteomics-based approach to investigate glycated/ glycosylated synovial fluid (SF) proteins and to identify molecular patterns potentially linked to joint degeneration in OADM.

**Methods:**

We performed the first global proteomic analysis of phenylboronic acid (PBA)-enriched cis-diol–containing glycation/ glycosylation-associated proteins from knee SF samples of OA and OADM patients. Enriched proteins were fractionated by SDS-PAGE and analyzed by LC-MS/MS, followed by quantitative SWATH-MS and bioinformatic pathway analyses. Selected proteins showing increased abundance in OADM were evaluated in SF and paired serum samples from a larger cohort using ELISA, and their discriminatory performance was explored using receiver operating characteristic (ROC) curve analysis.

**Results:**

Three proteins, high-temperature requirement protein A1 (HTRA1), cathepsin G (CTSG), and alpha-1-acid glycoprotein 1 (AGP1), were consistently more abundant in OADM compared with OA. Functional annotation associated HTRA1 and CTSG with extracellular matrix remodeling and degradative processes, while AGP1 was linked to inflammatory responses. Exploratory ROC analyses in both SF and serum suggested that these proteins have potential utility in distinguishing OADM from OA within the studied cohort, with SF and serum levels showing significant concordance in OADM patients.

**Conclusions:**

These findings suggest an association between T2DM-related metabolic dysregulation, synovial protein glycation/ glycosylation, and cartilage degeneration in OADM. HTRA1, CTSG, and AGP1 emerge as clinically measurable proteins with potential biomarker relevance for distinguishing OADM from OA, which may support improved disease stratification in future studies. Collectively, the results provide molecular insight into the co-pathogenesis of OA and T2DM and highlight glycation/ glycosylation-associated pathways as candidates for future precision-based therapeutic investigation, with the potential to mitigate OA severity in patients with T2DM and improve joint health.

**Supplementary Information:**

The online version contains supplementary material available at 10.1186/s12967-026-07984-8.

## Background

Osteoarthritis (OA), a degenerative joint disease, causes joint stiffness and chronic pain and significantly impairs mobility. The disease predominantly affects the knees, hips, and hands. It is characterized by synovial inflammation, subchondral bone remodeling, and cartilage degradation [1]. The prevalence of the disease in the elderly population renders it a leading cause of disability worldwide. In 2020, the global burden of OA was estimated at approximately 595 million patients, with projections indicating a further increase by 2050 [2]. Another disease that affects millions globally is type 2 diabetes mellitus (T2DM), a metabolic disorder marked by insulin resistance and hyperglycemia [3]. Fundamentally, both OA and T2DM are multi-factorial diseases influenced by a combination of genetic, demographic, and lifestyle factors [4].

Recent studies highlight significant convergence and a strong relationship between OA and T2DM and suggest that the two conditions often co-exist (OADM condition), not merely by coincidence, but are underpinned by similar risk factors like obesity and advancing age, which lead to complicated health challenges and diminished quality of life [4, 5]. Obesity intensifies cartilage degradation by causing mechanical overload on weight-bearing joints and promoting inflammation [6]. In addition, it plays a key role in inducing insulin resistance, a major contributor to the development of T2DM, and metabolic disturbances that can lead to systemic inflammation [7], exacerbating both OA and T2DM. The intersection of these risk factors justifies the increase in their likelihood of co-occurrence, besides directing toward potential overlapping pathophysiological mechanisms.

OADM presents a complex clinical challenge with symptoms, such as pronounced joint inflammation, increased stiffness, elevated severity, rapid progression, and a higher susceptibility to disability compared to OA [8]. T2DM is associated with low-grade chronic inflammation and elevated pro-inflammatory cytokines that exacerbate OA symptoms [9]. Chronic hyperglycemia in T2DM is widely known to induce the accumulation of advanced glycation end products (AGEs). AGEs can cross-link with collagen in joint cartilage, which has been associated with increased stiffness, thus reducing elasticity and impairing joint functionality [10]. Moreover, they interact with their receptors (RAGE), triggering oxidative stress and systemic inflammation, which contribute to cartilage degradation and joint damage. Hyperglycemia and AGE accumulation, therefore, serve as critical links between T2DM and OA pathogenesis, working efficiently towards modifying extracellular matrix (ECM) proteins, disrupting cartilage homeostasis, and promoting synovial inflammation [1].

Emerging pieces of evidence, hence, point towards shared molecular mechanisms underlying OADM co-pathogenesis, including inflammation, altered glucose metabolism, and changes in ECM turnover [6, 11]. However, despite the proposed implication, the precise role of protein glycation/ glycosylation in OADM pathogenesis and its impact on the pathophysiological fundamentals of OADM remain largely unexplored and inadequately understood. Compounding the concern, T2DM has even been linked to poorer outcomes following joint replacement surgeries [12], underscoring the need for targeted management strategies in the T2DM population. The growing burden of OADM patients in the healthcare system, therefore, calls for research for early diagnosis and effective intervention of OADM.

**Fig. 1:**
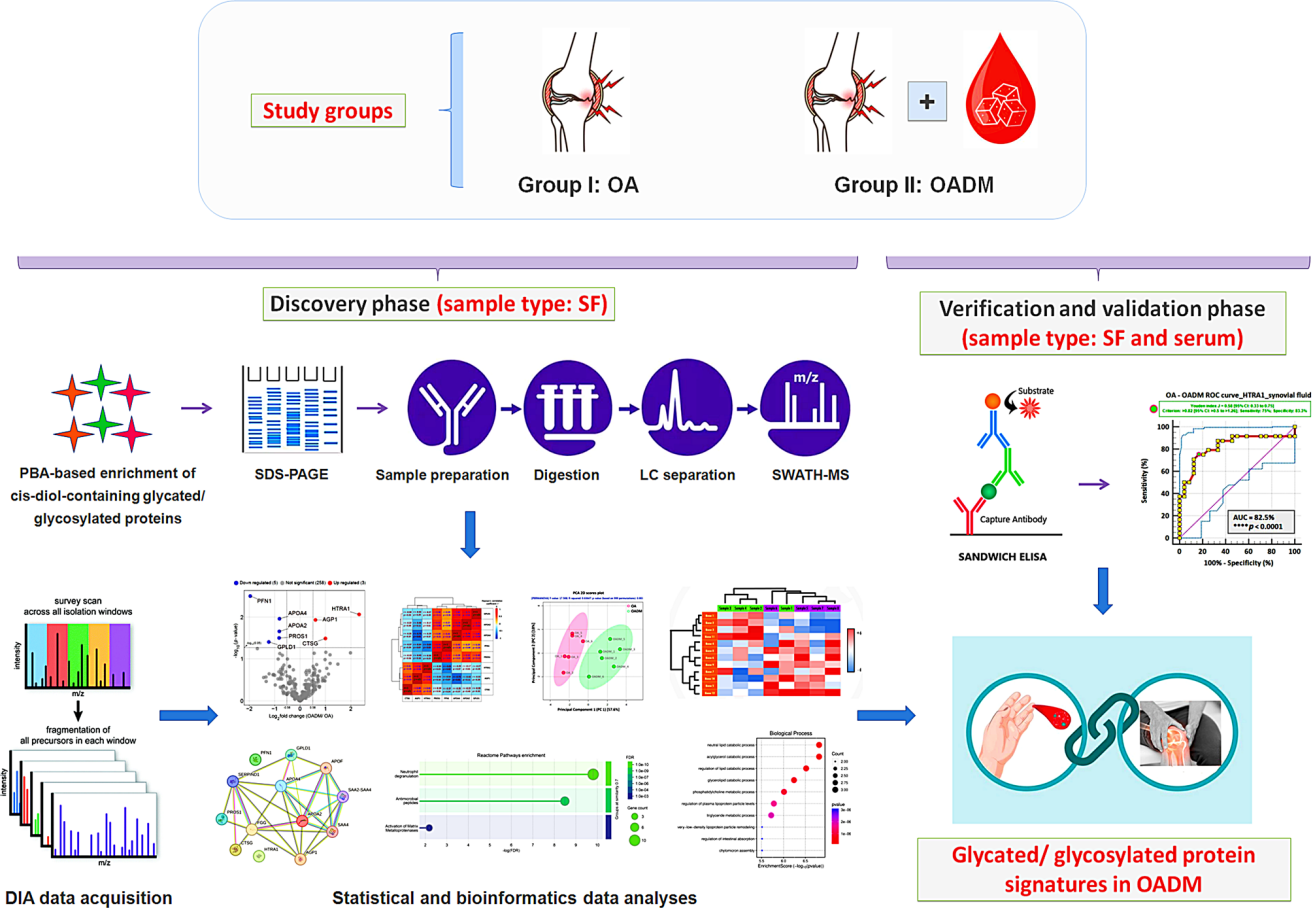
Overall workflow of the study. To investigate molecular features associated with joint degeneration in the co-morbid OADM condition, cis-diol-containing glycation/ glycosylation-associated SF proteins from OA and OADM patients were analyzed using a global proteomic approach. This was followed by bioinformatic pathway analyses and validation of key OADM-associated proteins in SF and serum

The present study aims to bridge the knowledge gaps by conducting a comparative global proteomic analysis of cis-diol-containing glycated/ glycosylated proteins enriched from synovial fluid (SF) of patients with OA and OADM, thereby identifying potential biomarkers and molecular pathways of relevance to OADM and informing strategies to improve patient outcomes (Fig. 1). Proteomics offers a powerful approach to understanding disease pathogenesis at the molecular level [13]. Since SF, the central nourishing medium and lubricant in joints, bathes synovium and the joint cartilage [14], we reasoned that it reflects the biochemical milieu and pathological state of the joint environment, and is an ideal bio-fluid for investigating OA-associated molecular changes. Studying variations in the SF proteome can hence reflect potential pathological mechanisms underlying OA, whilst providing insights into OA progression and its co-existence with diabetes.

## Methods

### Patient recruitment and study groups

The study was ethically approved by the Postgraduate Institute of Medical Education and Research (PGIMER), Chandigarh, India, Institutional Ethics Committee (IEC) vide no. IEC-12/2021–2238, and abides by the Declaration of Helsinki principles. The study subjects were recruited from the Outpatient Department (OPD) of Orthopedics, Postgraduate Institute of Medical Education and Research (PGIMER), Chandigarh, India, as per the following inclusion and exclusion criteria:

#### Patient inclusion criteria

Ethnic North Indian patients of either sex, aged 50–80 years, presented with OA-like symptoms such as intermittent or continuous pain in one or both knees for at least 3 months, and patients with OA along with T2DM, were recruited in the study. The severity of OA was assessed by radiologists and orthopedic consultants by visualizing the knee anteroposterior (AP) and lateral radiographs, followed by grading OA as per the Kellgren-Lawrence (KL) scale ranging from 0 to 4; grade 4 phenotype indicative of end-stage OA, marked by excessive sclerosis, definite deformity of bone ends, severe joint-space narrowing (bone-on-bone contact), and the presence of large osteophytes [15]. Confirmation of T2DM was made by evaluation of glycated hemoglobin (HbA1c) as a diagnostic test for T2DM as per the World Health Organization (WHO) expert consultation [16]; HbA1c > 6.5% corroborating to T2DM.

**Fig. 2:**
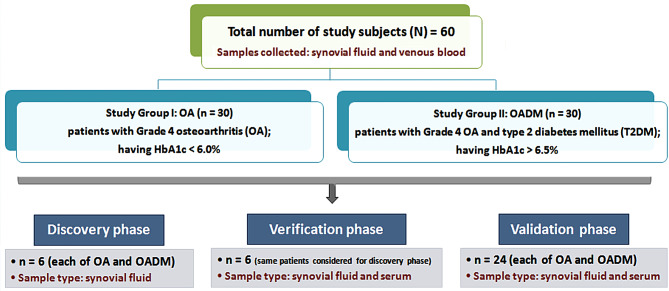
Flow diagram illustrating the study groups, alongside describing the assortment of study subjects. The figure outlines the number of patients recruited for the present study under the OA and OADM study groups. It also provides details of the type of samples collected and their utilization for different analysis phases of the research

The study groups for the present research were classified as: Group I (OA): 30 patients having Grade 4 OA and HbA1c < 6.0%, and Group II (OADM): 30 patients having Grade 4 OA and T2DM (HbA1c > 6.5%) (Fig. 2).

#### Patient exclusion criteria

Patients with a history of injury in knee joints, any previous knee surgeries, ligament injury, those diagnosed with osteoporosis, sarcopenia, or other bone disorders, or those suffering from musculoskeletal diseases like myopathies, polio, bone tumors, and osteomalacia, were excluded from the study. In addition, cancer patients, patients with a history of corticosteroid injections, those taking anti-tubercular drugs, anti-epileptic drugs, and drugs altering bone metabolism, and patients with pre-diabetic history (HbA1c = 6.0-6.5%) were also excluded.

### Sample collection, storage, and utilization

SF (1–2 mL) was collected from the affected knee joint of patients who underwent total knee replacement (TKR) surgery in the Orthopedics Operation Theater (OT), PGIMER. The collected fluid was centrifuged at 3,000$\times$g for 10 min, followed by separation and later storage of the supernatant at -80 °C until use. Blood (5 mL) was also withdrawn from the same patients, visiting Endocrinology and/or Orthopedics OPD, PGIMER, and recruited for the study. It was left undisturbed for 45 min, and centrifuged at 3,000$\times$g for 10 min to separate serum, followed by storage of the serum at -80 °C until use. Sample protein content was quantified using Pierce™ bicinchoninic acid (BCA) protein assay kit (Thermo Fisher Scientific, USA).

SF samples obtained from 6 patients in each study group, OA and OADM, were used for the discovery phase proteomic analysis. This cohort size was selected in accordance with the exploratory, hypothesis-generating design of the study and is consistent with previously published SF-based mass spectrometry proteomic investigations that prioritize deep proteome coverage and stringent candidate selection [14, 17]. For verification, both SF and paired serum samples from the same 6 patients per group were analyzed using orthogonal immunoassays to confirm key findings from the discovery phase. An expanded validation cohort comprising SF and paired serum samples from 24 patients per group was subsequently employed to assess the reproducibility and discriminatory potential of selected candidate proteins. A schematic overview of sample allocation across discovery, verification, and validation phases is provided in Fig. 2.

### Discovery phase analysis

#### Phenyl boronic acid (PBA)-based enrichment of cis-diol-containing glycated/ glycosylated proteins

Glycated/ glycosylated protein enrichment was performed by exploiting a principle based on phenyl boronate affinity chromatography. The 6 SF samples, considered for discovery phase analysis, were processed using NuGel™ phenyl boronic acid (PBA)-based glycoprotein enrichment kit (Biotech Support Group, USA), as per the manufacturer’s instructions. The processed eluate, comprising an enriched fraction of glycated/ glycosylated proteins, was collected, quantified for total protein content, and subsequently utilized for liquid chromatography-tandem mass spectrometry (LC-MS/MS) analysis.

#### Sodium dodecyl sulphate-polyacrylamide gel electrophoresis (SDS-PAGE)

The enriched SF fractions were electrophoresed on a 10% SDS-polyacrylamide gel to evaluate the extent of enrichment and to visualize the difference in protein bands between the enriched and the non-enriched samples. The gel was stained with Coomassie^®^ brilliant blue R-250 (HiMedia, India), followed by visualization using AlphaImager^®^ Mini gel documentation system (ProteinSimple, California, USA).

#### Sample preparation for LC-MS/MS

##### Protein precipitation, extraction, and estimation

Proteins in the samples were precipitated using chilled acetone, at -20 °C. Precipitated proteins were extracted by centrifugation at 15,000 rpm for 10 min at 4 °C. The pellets were dried at room temperature and subsequently resuspended in 50 mM ammonium bicarbonate (ABC) buffer (pH 7.8). Protein estimation was done by Bradford assay employing Bradford reagent [Merck (Sigma-Aldrich), USA; B6916] for which a bovine serum albumin (BSA) standard was prepared in ABC buffer, followed by measurement of the optical density (OD), at 595 nm, using a multimode plate reader (Infinite® M Plex, Tecan, Switzerland).

##### Protein de-glycosylation

The samples were deglycosylated using N-Glycosidase F (PNGase F) (Promega, USA; V4831) before LC-MS preparation. Fifteen µg of protein was diluted in 50 mM ABC buffer to a final volume of 16 µL. Subsequently, 1 µL PNGase F was added, and the reaction mixture was incubated at 37 °C for 4 h.

##### Protein reduction, alkylation, digestion, and desalting

Deglycosylated samples were further reduced with 1,4-dithiothreitol (DTT) (Sigma-Aldrich, USA), followed by alkylation using iodoacetic acid (IAA) (Sigma-Aldrich, USA). For DTT treatment, 2 µL 25 mM DTT was added to 4 µL sample, and incubated at 56 °C for 25 min. For the subsequent IAA treatment, 1 µL 55 mM IAA was added to 2 µL reaction mixture under dark conditions, and incubated at room temperature for 15 min.

Next, sample proteins were digested using MS-grade trypsin (Promega, USA; V5280) (enzyme concentration: 0.5 µg/µL in ABC buffer; ratio of sample: enzyme = 1: 20) to obtain peptides. The reaction mixture for trypsin digestion was incubated overnight at 37 °C. Enzyme activity was quenched by changing the pH using 50% formic acid (prepared in LC-MS water (Biosolve BV, The Netherlands; 232178). The samples were lyophilized using a SpeedVac (Thermo Fisher Scientific, USA) and stored at -20 °C until use. Peptides amounting to more than 5 µg were further subjected to desalting.

For desalting, all the reagents were strictly prepared in LC-MS water. Each dried sample was resuspended in 1 mL 0.1% formic acid. An HLB C18 column (Waters™ Corporation, USA) was then inserted into a 15 mL falcon tube. The column was activated by the addition of 1 mL 100% acetonitrile (ACN). It was subsequently re-equilibrated with 1 mL of 0.1% formic acid. Further, 1 mL digested sample was added to the column for peptide binding. The column was washed using 5% methanol. The washed column was transferred to a fresh 15 mL Falcon tube. 1 mL elution buffer, comprising 70% ACN, was utilized for the elution of peptides. Each step of desalting was ended by centrifugation of the mixture at 2,000$\times$g for 5 min. The eluted peptides were collected in fresh centrifuge tubes and lyophilized using a SpeedVac for 4 h, then stored at -20 °C until further use.

#### High-resolution sequential window acquisition of all theoretical mass spectra (SWATH-MS) analysis

The processed SF samples were analyzed for glycated/ glycosylated proteome at the Council of Scientific and Industrial Research - Institute of Genomics and Integrative Biology (CSIR-IGIB), New Delhi, India. Digested, desalted, and dried samples were resuspended in 0.1% formic acid, centrifuged at 12,000 rpm for 5 min, and transferred to LC vials for label-free MS/MS analysis. SWATH-MS was performed on a quadrupole time-of-flight (TOF) hybrid mass spectrometer (TripleTOF^®^ 6600, AB Sciex, USA) coupled to a nanoLC-425 system (Eksigent, Sciex, USA), operated in a data-independent acquisition (DIA) mode. Ion source parameters were optimized as follows: ion spray voltage 5.5 kV; curtain gas 25 psi; nebulizer gas 20 psi; source temperature 250 °C.

For each sample, 4 µg peptides were first loaded on a trap-column (ChromXP™ C18-CL, 5 μm, 120 Å, Eksigent) and desalted online at 10 uL/min for 10 min. Peptides were subsequently resolved on a reverse-phase C18 analytical column (ChromXP™ C18, 3 μm, 120 Å, Eksigent) using a 55 min gradient of buffer A (0.1% formic acid in LC-MS water) and buffer B (0.1% formic acid in ACN), at a flow rate of 5 µL/min. For the gradient, buffer A was gradually decreased from 97% to 75% in the first 38 min. It was decreased to 68% in the next 5 min, to 20% within 2 min, and further to 10% post 30 s. It was held at 10% concentration for 2.5 min. The concentration of buffer A was then increased to that of the initial 97% in 1 min, followed by reconditioning of the column for 6 min, and lastly, its washing for 2 min before the next run.

For acquiring data concerning mass-to-charge ratios (m/z) of ions in the samples, Analyst^®^ TF v1.7.1 software (Sciex, USA) was used using optimized source parameters. MS survey scans were acquired over an m/z range of 400–1250 with an accumulation time of 0.25 s, while MS/MS scans were acquired over an m/z range of 100–1500 with an accumulation time of 0.025 s. No rolling collision energies were applied.

The MS output data of two formats, .wiff, which contains metadata associated with MS data acquired using the AB Sciex instrument, and .wiff.scan, which stores the actual x, y scan data representing the m/z ratio and intensity of the detected ions, were deposited to the ProteomeXchange consortium (http://proteomecentral.proteomexchange.org) *via* the PRoteomics IDEntification database (PRIDE) partner repository [18]. The files can be accessed with the dataset identifier PXD062955.

##### Spectral library matching and data processing

Mass spectra acquired by SWATH-MS in DIA mode were matched against an in-house SF-specific spectral library comprising 355 proteins. This library was previously generated at CSIR-IGIB, New Delhi, India, from human SF samples using strong cation exchange (SCX) fractionation followed by LC-MS/MS analysis. The deliberate use of a SF-specific library was intended to enhance biological relevance and analytical reliability, as it provides representative coverage of key synovial proteins implicated in OA and OADM, including ECM-associated proteins, proteases, inflammatory mediators, metabolic regulators, and acute-phase proteins, thereby supporting robust and biologically meaningful comparative proteomic analyses.

Spectral matching and peptide extraction were performed using PeakView v2.2 software (AB Sciex, USA), followed by quantitative peak area extraction using MarkerView™ v1.2.2 software (Sciex, USA). SWATH retention time calibration method of the PeakView software was applied to ensure consistent peptide identification and quantification across runs. The peptide query parameters (PQPs) for peak extraction were set to default values, except as specified. False discovery rate (FDR) was set to 1%. Other processing settings were as follows: number of peptides per protein: 10; number of transitions per peptide: 5; peptide confidence threshold: 95%; modified peptides: excluded; extracted ion chromatogram (XIC) window: 55 min; XIC width: 75 ppm. Protein abundances were estimated by quantification of the total peak area under the curve (AUC) defined by the intensity peaks of respective peptides. The protein abundances were further compared between samples.

In parallel, an additional MS data analysis was performed using MaxQuant v2.4.13 software. However, downstream analyses prioritized identification and quantification results obtained using the in-house SF-specific SCIEX spectral library, as this approach offered greater contextual relevance to joint biology compared with searches against the general *Homo sapiens* reference proteome (UniProt Proteome ID: UP000005640).

#### Statistical analyses of SWATH-MS data

Total area sum (TAS) intensity-based data normalization, for internal correction and adjustment of anomalies, was carried out using MarkerView software. Data was then exported to a Microsoft Excel (XLSX) spreadsheet. It was log2 transformed to account for naturally skewed intensity values. Log2 fold change (log2FC) values for each protein were calculated by subtracting the mean of log2 values of control cases (Group I: OA) from those of test cases (Group I: OADM). Fold changes were subsequently derived using base-2 exponentiation of log2FC values.

##### Bivariate analyses

A two-sample equal variance Student’s t-test following a two-tailed distribution was applied to the normalized and log2 transformed data in an XLSX worksheet. Proteins exhibiting a *p*-value < 0.05 were filtered as significant. Further segregation, considering a log2FC cut-off of ± 0.58, categorized the data into significantly differentially abundant proteins. Statistically significant differences between the two datasets were illustrated by a scatter volcano plot drawn on the Scientific and Research plot (SRplot) tool (http://www.bioinformatics.com.cn/SRplot) [19], along with the violin plots generated using MetaboAnalyst v6.0 web-based platform (https://www.metaboanalyst.ca/).

Next, Pearson’s correlation was conducted to examine the probability and the degree of relationship between the significantly differentially abundant PBA-enriched cis-diol-containing glycated/ glycosylated proteins, using MetaboAnalyst v6.0. Pearson’s correlation coefficient ‘*r*’ was calculated by considering the covariance of two proteins at a time and dividing it by the product of their standard deviations. Further, hierarchical relationships between proteins were elucidated by exploiting Pearson’s *r* distance measures. The clustering patterns were displayed using dendrograms.

##### Multivariate analyses

The dimensionality of the significant data generated by SWATH analysis was reduced by exploiting an unsupervised machine learning (ML) algorithm called principal component analysis (PCA) that considers all factors, smartly combines them, and identifies a set of orthogonal axes, called PCs, to capture maximum variance in the data. PCA, however, excluded the consideration of group labels. The ordination analysis was used to transform high-dimensional data into a lower-dimensional subspace while retaining as much information as possible. PCA was performed to examine interrelations among samples from different datasets, using MetaboAnalyst v6.0. Normalized and log2-transformed significant data were input into the server, checked for data integrity, and proceeded for PCA. Statistical significance of the group patterns was evaluated using PERmutational Multivariate ANalysis of VAriance (PERMANOVA), which compared the variation between OA and OADM groups, to the variation within groups.

Next, a heatmap was constructed using MetaboAnalyst v6.0 to represent the varying abundance of individual significant proteins in all the samples. *Z*-score standardization was performed on the previously normalized and log2-transformed protein abundances. For this, the features (proteins) were standardized by the autoscale function. Varying color intensities representative of the *Z*-scores were used to highlight the proteins that were differentially abundant in the two study groups. *Z-*score-scaled proteins were further subjected to an unsupervised multivariate analysis technique called hierarchical cluster analysis (HCA). HCA employed the Average linkage method based on Euclidean distances to hierarchically cluster proteins with similar trends in abundance between the samples.

#### Bioinformatics analyses of differentially abundant proteins

The statistically significantly differentially abundant proteins were further utilized for bioinformatics analyses to investigate the molecular pathways underlying the co-pathogenesis of the OADM condition.

##### Functional and pathway enrichment analysis

Pathway analyses, including Gene Ontology (GO) and Kyoto Encyclopedia of Genes and Genomes (KEGG) pathway mapping, were done by exploiting R software in the SRplot tool that integrates the R package clusterProfiler and the R/Biocondutor package pathview. Human (human, has) was chosen as the species for GO/ pathway enrichment analysis. For more defined observations, two sets of analyses were run on the significantly differentially abundant proteins, one considering the upregulated elements while the other taking into account the proteins downregulated in patients with OADM, when compared to those having OA.

Additionally, the DAVID resource system (https://davidbioinformatics.nih.gov/home.jsp) [20] was screened to gain deeper insights into the biological functions of abundant determinants identified in the study. The comprehensive knowledge base was used to verify the GO and KEGG pathways obtained from SRplot and enhance the interpretation of findings to incorporate reliable functional annotations into our results. A list of genes corresponding to the abundant proteins was uploaded to the DAVID database and submitted to the Gene ID conversion tool for conversion of Gene IDs to UniProt Accessions. It was screened against the species *Homo sapiens*. The converted list was then uploaded to DAVID as a Gene List and analyzed using the DAVID functional annotation tool. The gene reports were also searched for detailed literature analysis to elucidate the linkage of particular proteins with diseases.

##### Construction of protein-protein interactome

Search Tool for the Retrieval of Interacting Genes/ Proteins (STRING) tool v.12.0 (https://string-db.org/) was used for generating networks of the significantly differentially abundant proteins in the OADM condition based on known and predicted protein-protein interactions (PPIs). This was done to identify key players in OADM co-pathogenesis and gain an understanding of the functional relationships among the associated proteins. FASTA sequences of the shortlisted proteins were input into the STRING database, first collectively, and then only the upregulated ones, and screened against *H. sapiens* as the organism of interest. The generated interactome was visualized as a graph following FDR stringency of 0.05, while adjusting the confidence score to 0.4. Reported FDR values corresponded to *p*-values adjusted for multiple hypothesis testing using the Benjamini-Hochberg correction method. STRING tool was additionally explored for Reactome pathway database-based functional enrichment analysis of the interacting proteins.

### Verification and validation phase analysis

#### Sandwich immunoassay: Enzyme-linked immunosorbent assay (ELISA)

The top three differentially upregulated proteins were chosen for verification and validation of SWATH-MS data by ELISA. Sandwich ELISA was performed to detect the presence and quantity of the respective proteins in patient SF, as well as serum samples. ELISA kits for HtrA serine protease 1 (HTRA1) (Cat. No. E-EL-H0423) and alpha-1-acid glycoprotein 1 (AGP1) (Cat. No. E-EL-H0001) were procured from Elabscience^®^ (Elabscience Bionovation Inc., USA), whereas, that for cathepsin G (CTSG) (Cat. No. EH1903) was acquired from FineTest^®^ (Fine Biotech Co. Ltd., Wuhan, China). The coefficient of variation (CV, %) for intra-assay precision (low, medium, and high concentration samples tested 20 times on the same plate) and inter-assay precision (the same samples tested 20 times across three different plates) was < 10% for all ELISA kits used for the three proteins, in accordance with the manufacturers’ instructions.

#### Statistical analyses of ELISA data

##### Regression model for standard curve generation and data analysis

Optical density (OD) readings, averaged from duplicates for each standard and sample, were plotted using the Quest Graph™ four-parameter logistic (4PL) curve calculator (AAT Bioquest, Inc.; https://www.aatbio.com/tools/four-parameter-logistic-4pl-curve-regression-online-calculator). Analyte concentrations were calculated by interpolating sample OD values from the corresponding standard curves and adjusted for dilution factors.

##### Data normality testing and graphing

Data normality was evaluated using both Shapiro-Wilk and Lilliefors-corrected Kolmogorov-Smirnov tests, performed using Statistical Product and Service Solutions (SPSS) v20 software (SPSS 20, IBM^®^ Corp. Armonk, New York, USA). Normally distributed data were analyzed by parametric two-tailed Welch’s t-test for significance and are presented as Mean ± standard deviation (SD). Non-normal (skewed) or mixed-distribution data were subjected to a two-tailed non-parametric Mann-Whitney test for significance, and are presented as Median (IQR: interquartile range), where, IQR = Q_3_ – Q_1_. If one group satisfied normality assumptions while the other did not, the combined dataset was treated as non-normal and analyzed accordingly. All statistical analyses and graphical representations were performed using GraphPad Prism v8.0.2 (GraphPad Software, Inc., USA).

##### Correlation analyses of paired SF and serum levels in OADM validation cohort

To evaluate the translational relationship between local joint-associated protein levels and systemic abundance, exploratory correlation analyses were performed between paired SF and serum concentrations of the validated proteins. Correlation analyses were restricted to the OADM validation cohort (*n* = 24), in which paired SF and serum samples were available for each subject. Considering the non-normal distribution of ELISA-derived variables, Spearman’s rank correlation was applied to assess monotonic associations between SF and serum levels of HTRA1, CTSG, and AGP1. Two-tailed *p*-values were calculated, and correlations were considered statistically significant at *p* < 0.05. Correlation analyses were performed using GraphPad Prism v8.0.2 (GraphPad Software, Inc., USA). Acknowledging the exploratory nature of the study, correlation results were interpreted as hypothesis-generating and were not adjusted for multiple comparisons.

##### Evaluating the diagnostic performance of key proteins by receiver operating characteristic (ROC) curve analysis

ROC curve analysis was performed to exploratorily evaluate the discriminatory performance of shortlisted and ELISA-validated differentially abundant proteins in distinguishing OADM from OA cases, in the validation cohort (*n* = 24). ROC analyses were conducted separately for SF and paired serum samples using MedCalc^®^ statistical software v20 (MedCalc Software Ltd., Ostend, Belgium; https://www.medcalc.org; 2021). The area under the ROC curve (AUC) was calculated as a summary measure of classification performance, and statistical significance was assessed using the non-parametric Mann-Whitney *U* test [21]. To account for uncertainty arising from the limited sample size, ROC/ AUC values were accompanied by bias-corrected and accelerated (BC_a_) 95% confidence intervals (CIs), estimated using bootstrap resampling (1000 iterations). Further, the Youden index *J* was calculated to identify the optimal operating point for each ROC curve, defined as the criterion value that maximized the sum of sensitivity and specificity when equal weight was assigned to both parameters. $J=\mathrm{max}\{{sensitivity}_{c}+{specificity}_{c}-1\}$, where *J* is the maximum vertical distance between the ROC curve and the diagonal line, and *c* ranges over all possible criterion values. It equals 0 for a test with poor diagnostic accuracy and 1 for a perfect test. Corresponding cut-off values, sensitivity, and specificity estimates were derived at this point. The obtained AUC was lastly converted to a percentage value, AUC 100% representing 100% accuracy of the marker in correctly differentiating between OA and OADM cases. Given the exploratory nature of the study and the small cohort, ROC analyses were performed to estimate discriminatory trends rather than to establish definitive diagnostic thresholds, and all performance metrics were interpreted accordingly.    

## Results

### Demographic and laboratory parameter analysis of OA and OADM study subjects

SF and blood samples were collected and utilized for the discovery, verification, or validation phase of analysis, as depicted in Fig. 2. The demographics and laboratory characteristics of recruited patients are provided in Table 1 and Additional Fig. 1a and b. The cumulative fasting blood sugar (FBS) and the HbA1c levels of samples from the OA and OADM categories appeared significantly divergent. However, similarities were observed between demographic profiles of the subjects, such as age, sex, and body mass index (BMI). Furthermore, no appreciable variations were noted for the average total protein concentration of both SF and serum samples from the two study groups. In addition, clinically diagnosed hypertension (≥ 130/80 mmHg) and hypothyroidism were documented as co-morbid conditions across both study groups, with a higher prevalence of hypertension observed in OADM compared with OA in the validation cohort, while hypothyroidism was present in a smaller subset of patients in both cohorts (Table 1). These comorbidity distributions are reported to provide additional clinical context for the study population. Statistical analyses presented in Table 1 were performed as described under the ‘Data normality testing and graphing’ subsection of the ‘Verification and validation phase analysis’ in the Materials and Methods.

**Table 1:** Details of demographics and laboratory parameters of patients, enrolled in different study groups, and considered for the discovery, verification, and validation phase analysis. Data normality was formally assessed using both the Shapiro-Wilk and the Lilliefors-corrected Kolmogorov-Smirnov tests. Normal data (#) were analyzed by parametric two-tailed Welch’s t-test and are presented as Mean ± SD, whereas skewed or mixed-distribution data (###) were evaluated using non-parametric Mann-Whitney test and are shown as Median (IQR). Comparisons were made between the two study groups, OA and OADM. *p < 0.05, **p < 0.01, and ****p < 0.0001 represent statistically significant data, while ns indicates non-significant data

Characteristics	Analysis phase	Group I: OA	Group II: OADM	*p*-value
Sample size (n)	Discovery/ Verification	6	6	*-*
	Validation	24	24	*-*
Age (years) ^#^ (Mean of years ± SD)	Discovery/ Verification	68 ± 5.66	68.67 ± 6.65	0.8558 ^ns^
	Validation	65.63 ± 7.17	65.67 ± 8.19	0.9851 ^ns^
Sex [% females (n): % males (n)]	Discovery/ Verification	83% (5): 17% (1)	83% (5): 17% (1)	-
	Validation	96% (23): 4% (1)	79% (19): 21% (5)	-
Body mass index (BMI) (kg/m^2^) ^###^ Median (IQR: Q_3_ – Q_1_)	Discovery/ Verification	26.00 (29.83–25.80)	26.95 (28.48–25.55)	0.7479 ^ns^
	Validation	26.85 (29.23–25.65)	27.50 (31.68–25.73)	0.5494 ^ns^
WHO classification of body weight based on BMI (Underweight: Normal: Overweight: Obese Class I: Obese Class II: Obese Class III)	Discovery/ Verification	0: 0: 5: 1: 0: 0	0: 1: 5: 0: 0: 0	-
	Validation	1: 4: 14: 2: 1: 2	1: 3: 12: 7: 1: 0	-
Fasting blood sugar (FBS) (mg/dL) ^#^ (Mean levels ± SD)	Discovery/ Verification	101.33 ± 15.55	156.67 ± 40.70	0.0208 *
	Validation	97.96 ± 10.21	153.70 ± 40.39	< 0.0001 ****
Glycated hemoglobin (HbA1c) (%) ^###^ Median (IQR: Q_3_ – Q_1_)	Discovery/ Verification	5.50 (5.78–5.25)	6.95 (7.72–6.80)	0.0048 **
	Validation	5.50 (5.78–5.43)	6.90 (7.38–6.73)	< 0.0001 ****
Total protein concentration (mg/mL) ^#^ (Mean concentration ± SD)	Discovery/ Verification	SF: 29.97 ± 7.09	SF: 31.59 ± 7.68	0.7122 ^ns^
		Serum: 59.67 ± 1.48	Serum: 61.25 ± 2.28	0.1876 ^ns^
	Validation	SF: 34.88 ± 8.72	SF: 36.74 ± 6.44	0.4048 ^ns^
		Serum: 60.32 ± 6.13	Serum: 61.86 ± 3.70	0.2988 ^ns^
Clinically diagnosed hypertension (≥ 130/80 mmHg) [% Yes (n): % No (n)]	Discovery/ Verification	83% (5): 17% (1)	67% (4): 33% (2)	-
	Validation	54% (13): 46% (11)	75% (18): 25% (6)	-
Clinically diagnosed hypothyroidism [% Yes (n): % No (n)]	Discovery/ Verification	50% (3): 50% (3)	33% (2): 67% (4)	-
	Validation	17% (4): 83% (20)	21% (5): 79% (19)	-

### Global proteome profiling facilitated the identification of abundant glycated/ glycosylated proteins in SF of patients with OA and OADM

**Fig. 3:**
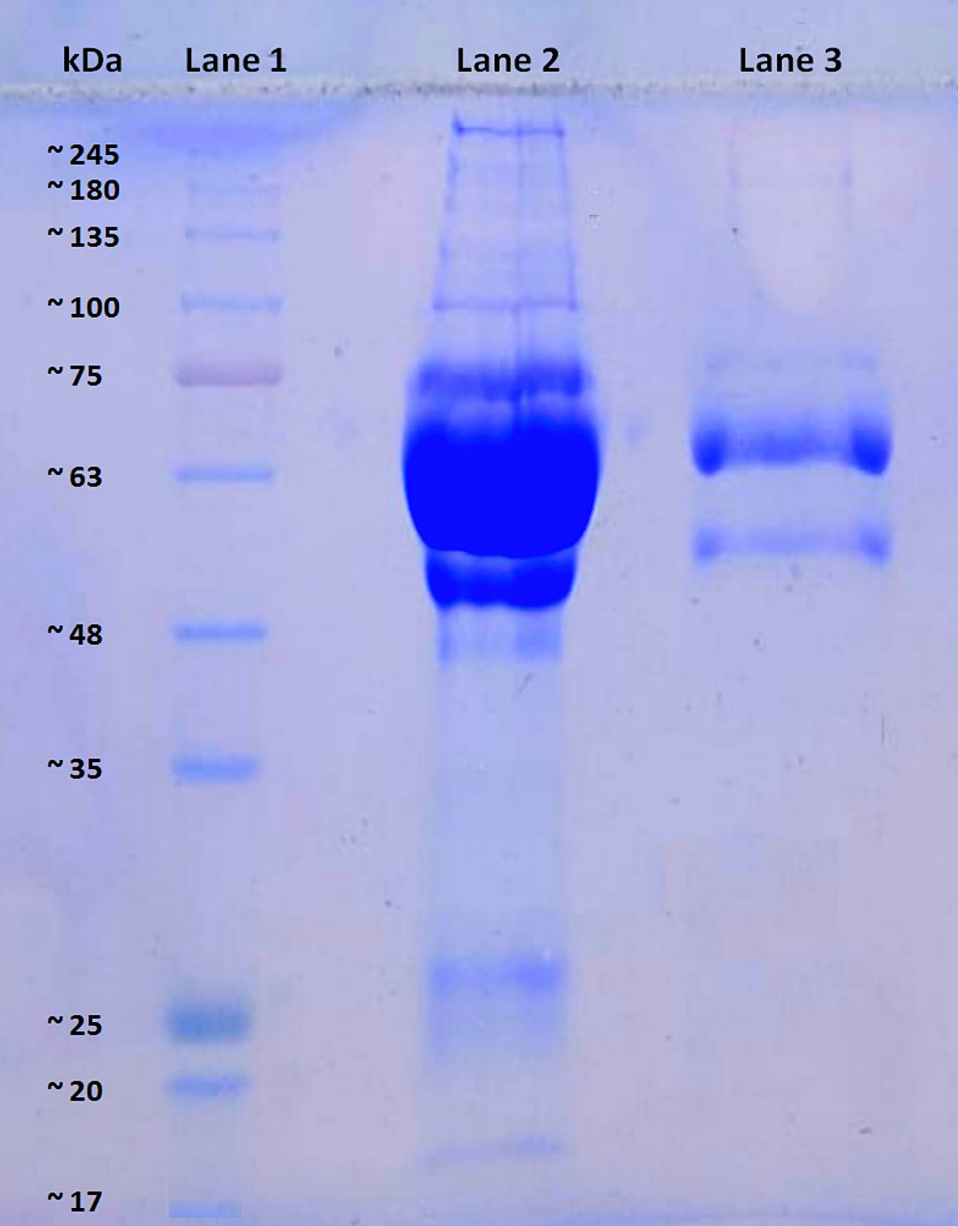
SDS-PAGE analysis of a synovial fluid (SF) sample following PBA-based enrichment. Representative SDS-PAGE image of an SF sample after enrichment of cis-diol-containing glycation/ glycosylation-associated proteins. Electrophoresis was performed on a 10% SDS-polyacrylamide gel. Lane 1 shows the protein molecular weight marker (HiMedia, India; Cat. No. MBT092), lane 2 represents the non-enriched SF sample, and lane 3 depicts the SF sample enriched using the NuGel™ PBA-based glycoprotein enrichment kit (Biotech Support Group, USA). Protein loading in lanes 2 and 3 was 10 µg each

Specificity of the PBA ligand yielded enriched heterogeneous sets of 1,2-cis-diol-containing glycated/ glycosylated proteins. Figure 3 portrays the SDS-PAGE image showing protein bands of a SF sample before and after being enriched for the desired fraction of proteins. The enriched fraction appeared with glycated/ glycosylated proteins unmasked from high-abundance proteins, most notably albumin (molecular weight = 66.5 kDa).

**Fig. 4:**
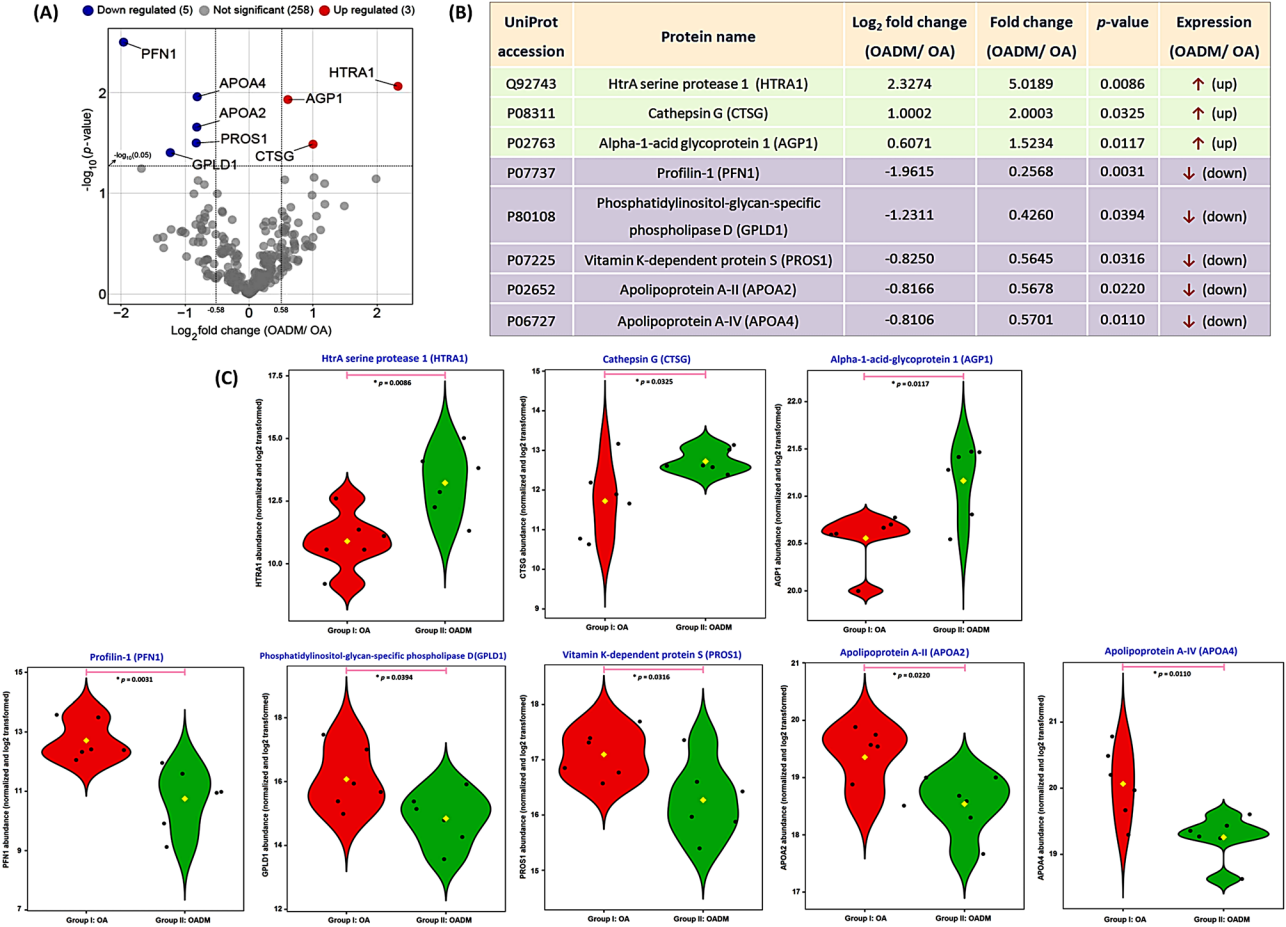
Identification of differentially abundant SF proteins in OADM. (**A**) Volcano plot illustrating the relationship between magnitude of change (log₂ fold change) and statistical significance (*p*-value) for 266 proteins identified by SWATH-MS analysis. A stringent FDR threshold of 1% was implemented during spectral matching and peptide extraction to ensure high confidence in protein identification. SWATH-MS data were subsequently normalized using total area sum (TAS) normalization. The normalized and log₂-transformed protein abundances were compared between OA and OADM groups using a two-tailed Student’s t-test. Proteins meeting the significance thresholds of *p* < 0.05 and |log₂FC| ≥ 0.58 were considered differentially abundant. (**B**) List of significantly upregulated and downregulated PBA-enriched cis-diol-associated proteins identified based on the applied log₂FC cut-off. (**C**) Violin plots depicting the distribution of normalized and log₂-transformed abundances of selected differentially abundant proteins across OA and OADM groups. Black dots represent individual sample values, while yellow dots indicate mean protein abundance. Kernel width reflects the relative density of data points. For each analysis, an asterisk (*) denotes *p*-value < 0.05

The enriched fractions were further processed and run through SWATH-MS. Analysis of SWATH-MS data resulted in the identification of 266 proteins at 1% peptide FDR, among the OA and OADM study groups (Additional Table 1). The statistically significant proteins (*p*-value < 0.05) were shortlisted and categorized into differentially abundant cis-diol-containing glycated/ glycosylated proteins based on a log2FC cut-off value of ± 0.58 (Fig. 4A). Proteins with log2FC > + 0.58 (FC > 1.5) corroborated to proteins upregulated in the OADM disease condition [high-temperature requirement serine protease A1 (HTRA1), cathepsin G (CTSG), and alpha-1-acid glycoprotein 1 (AGP1)], while those with log2FC < -0.58 (FC < 0.67) endorsed downregulated proteins [profilin-1 (PFN1), phosphatidylinositol-glycan-specific phospholipase D (GPLD1), vitamin K-dependent protein S (PROS1), apolipoprotein A-II (APOA2), and apolipoprotein A-IV (APOA4)] (Fig. 4B). To gather a clearer understanding of the distinctions, the distribution of the normalized and log2-transformed numeric data, representative of the abundance of these proteins among the two study groups, was depicted using violin plots (Fig. 4C).

### Strong correlations indicated similar expression patterns and unraveled functional relationships between upregulated or downregulated proteins

**Fig. 5:**
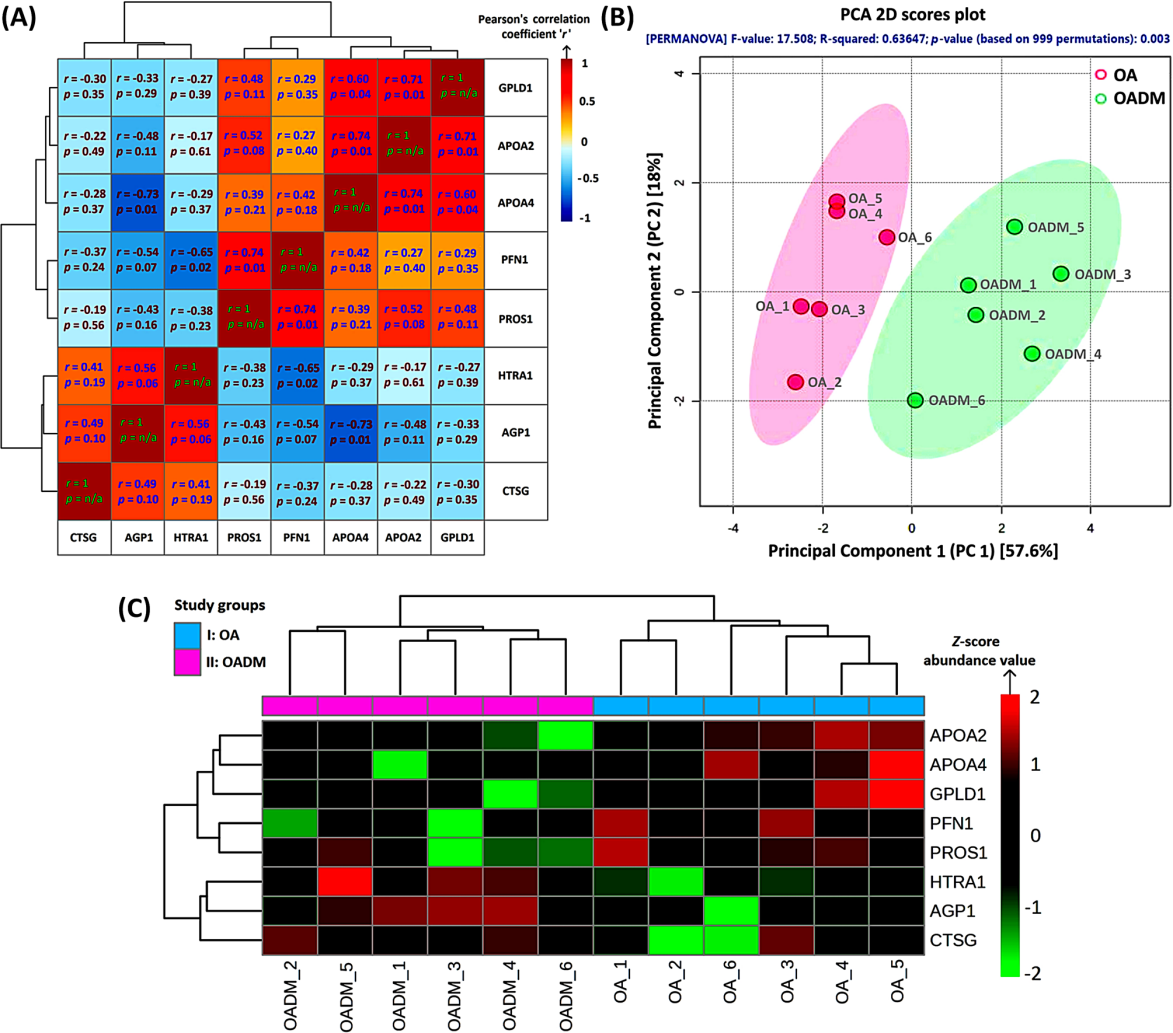
Correlation, unsupervised multivariate analysis, and clustering of glycation/ glycosylation-associated SF proteins in OADM. (**A**) Pearson’s *r* correlation heatmap depicting pairwise relationships among significantly differentially abundant proteins. Correlation coefficients (*r*) range from − 1 to + 1 and are represented by a fixed color scale, with positive and negative values indicating direct and inverse relationships, respectively. Only correlations with *p* < 0.05 were considered statistically significant. Proteins were hierarchically clustered and reordered based on similarity in correlation patterns. *p*-value was computed using one-way ANOVA. (**B**) PCA 2D scores plot illustrating sample-wise variation based on the abundance of differentially abundant PBA-enriched cis-diol-associated proteins. Each point represents an individual SF sample, summarizing the contribution of all quantified proteins, while shaded ellipses indicate the 95% confidence intervals for each study group. Clear separation between OA and OADM samples was observed, reflecting distinct SF proteomic profiles. Group separation was statistically assessed using PERMANOVA (*p*-value based on 999 permutations = 0.003), based on Euclidean distance with 999 permutations (*n* = 6 per group). (**C**) Heatmap showing hierarchical clustering of samples (columns) and significant proteins (rows) based on *Z*-scored, normalized, and log₂-transformed protein abundances. The color scale represents *Z*-scores, indicating the number of standard deviations by which the abundance of a given protein deviates from its mean value across all samples. A *Z*-score of 0 indicates abundance equal to the mean. Positive *Z*-scores denote relatively higher protein abundance, whereas negative *Z*-scores indicate lower-than-average abundance. Dendrograms were generated using Euclidean distance and Average linkage clustering

The strength and direction of the linear relationship between the significant proteins were measured by Pearson’s correlation coefficient ‘*r*’. Correlation heatmap showed that there was a statistically significant positive relationship between APOA4 and APOA2 (*r* = 0.74; *p* = 0.01), PROS1 and PFN1 (*r* = 0.74; *p* = 0.01), APOA2 and GPLD1 (*r* = 0.71; *p* = 0.01), and APOA4 and GPLD1 (*r* = 0.60; *p* = 0.04). A positive correlation indicated that an increase in the abundance of one protein was related to a simultaneous increase in the abundance of the other protein. Furthermore, a statistically significant negative relationship was also observed between AGP1 and APOA4 (*r* = -0.73; *p* = 0.01), and HTRA1 and PFN1 (*r* = -0.65; *p* = 0.02) (Fig. 5A). A negative correlation indicated that an increase in the abundance of one protein was related to a simultaneous decrease in the abundance of the other protein. Overall, the correlation plot revealed that expression patterns of the upregulated or downregulated protein subsets were similar, and the proteins in these subsets were linearly related.

### Unsupervised multivariate analysis delineated variations across study groups and revealed key cis-diol-containing glycation/ glycosylation-associated protein signatures in OADM

PCA was performed to reduce potential bias in data clustering and discern maximum data variance between OA and OADM study groups, as displayed by two PCs, PC1 and PC2, which collectively explain 75.6% of the accumulated variance. PC1 demonstrated a strong association with the variations within sample categories, effectively capturing the core source of variance in the datasets. PCA showed that the OA and OADM samples clustered distinctly, indicating a pronounced dissimilarity among the proteome profiles of SF samples in both groups (Fig. 5B). This was confirmed by the significance of PERMANOVA (*p*-value based on 999 permutations = 0.003), with an F-value of 17.508. The high R-squared value of 0.64 further indicates that PCA explained a huge variation.

Further, the heatmap showed that the significant proteins that were differentially abundant among the two study groups were clustered based on whether they were upregulated or downregulated. The HCA-based rearrangement of the rows and columns placed similar samples and proteins closer (Fig. 5C). The grouping is directed towards identifying the biological signatures associated with the OADM condition, alongside providing evidence of their similar behavior across sample clusters and potential regulation by common pathways. Three proteins, HTRA1, CTSG, and AGP1, were identified as the key glycated/ glycosylated signatures of the OADM disease condition.

### Pathway enrichment analyses (GO and KEGG) uncovered molecular processes associated with the differentially abundant proteins

**Fig. 6:**
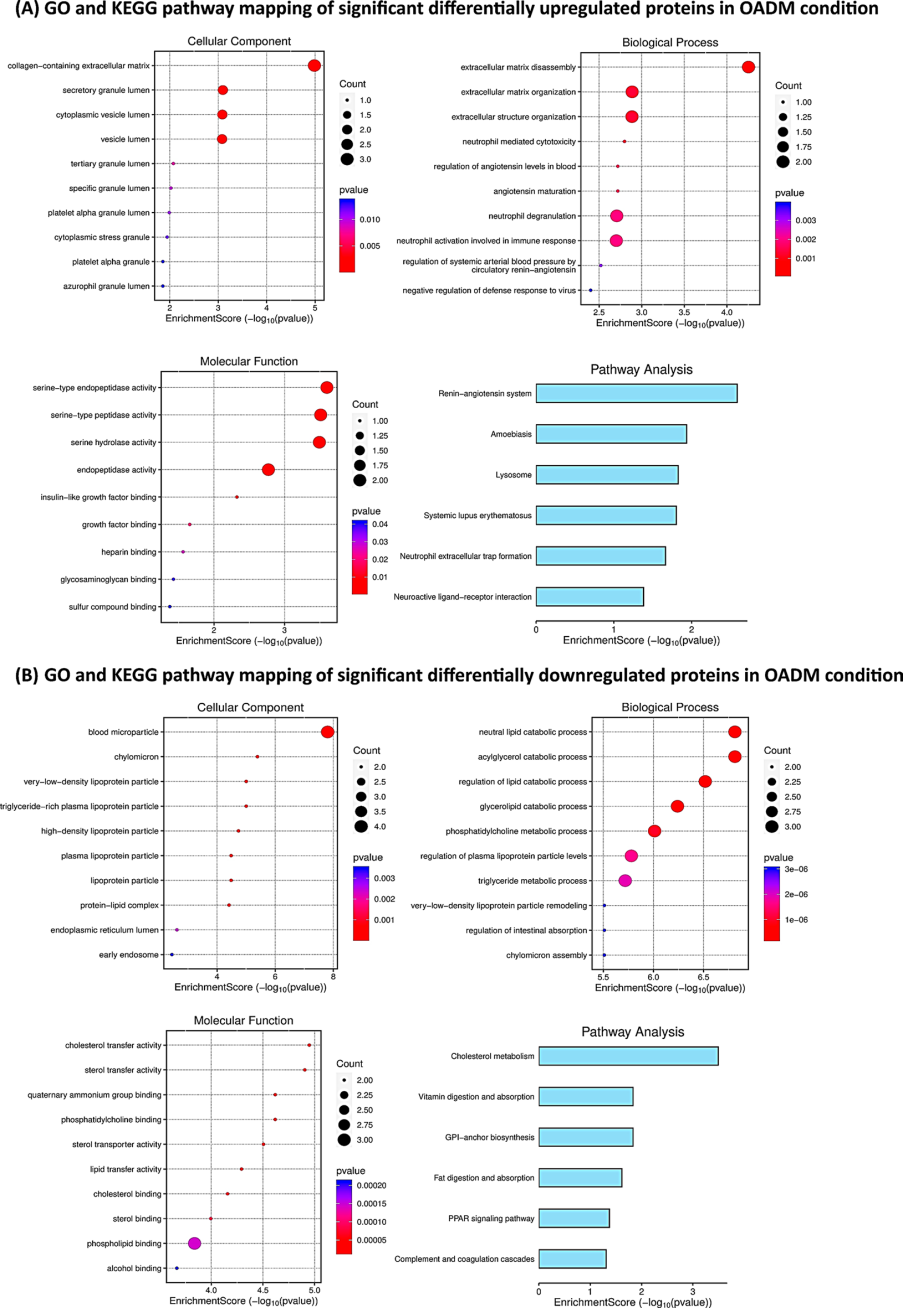
Functional pathways associated with SF proteomic alterations in OADM. (**A**) GO and KEGG pathway plots of significantly differentially upregulated proteins in OADM condition. (**B**) GO and KEGG pathway plots of significantly differentially downregulated proteins in OADM condition. In the bubble plots, dot size represents the number of differentially abundant proteins mapped to each enriched term (Count), while color intensity indicates the significance level (*p* < 0.05), calculated using a hypergeometric distribution

GO analysis of the significant differentially upregulated proteins in OADM condition revealed majority of the proteins with cellular component as collagen-containing ECM, biological processes as ECM disassembly, ECM organization, regulation of angiotensin levels in blood, angiotensin maturation, neutrophil degranulation, and regulation of systemic arterial blood pressure by circulatory renin-angiotensin, while molecular function as serine-type peptidase or hydrolase activity. In addition, the renin-angiotensin system (RAS) was decoded as the enriched KEGG pathway amongst the upregulated proteins (Fig. 6A). On the contrary, GO analysis of the significantly differentially downregulated proteins in the OADM condition linked the majority of the proteins with cellular components, such as blood microparticles, biological processes, including lipid, acylglycerol, and glycerolipid catabolism, and triglyceride metabolic process, while molecular function like sterol transfer activity. In addition, cholesterol metabolism, glycosylphosphatidylinositol (GPI)-anchor biosynthesis, fat digestion and absorption, peroxisome proliferator-activated receptor (PPAR) signaling pathway, complement, and coagulation cascades were identified as the enriched KEGG pathways amongst the downregulated proteins (Fig. 6B).

### STRING-derived interactome highlighted potential protein associations by leveraging both verified and projected interactions

**Fig. 7:**
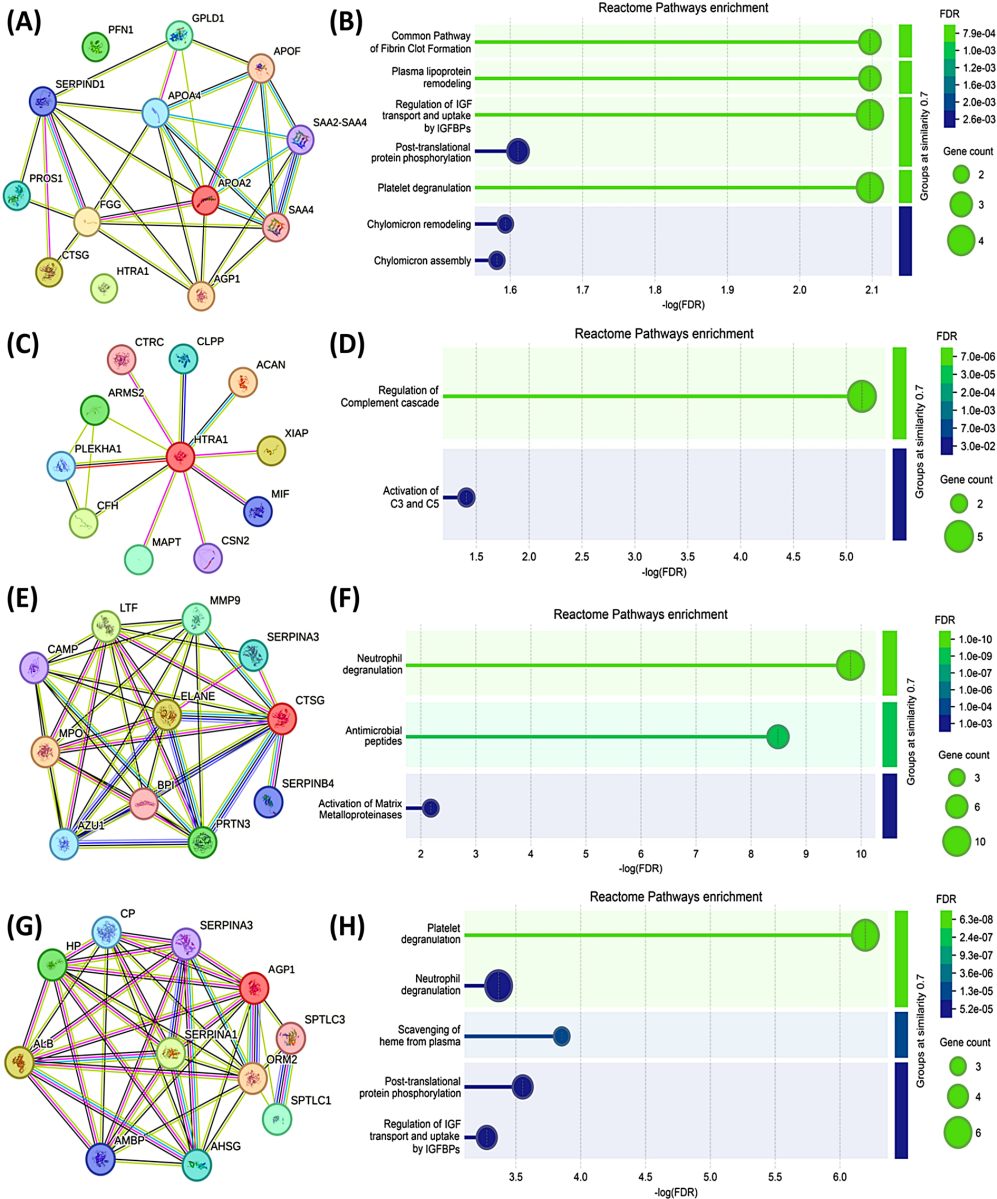
STRING protein-protein interaction (PPI) networks and Reactome pathway enrichment analyses OADM-associated SF protein signatures. (**A**) Integrated PPI network (interactome) of the significantly differentially abundant proteins in the OADM condition, and (**B**) reactome pathways enrichment of the collective network. (**C**) Interactome centered on HtrA serine protease 1 (HTRA1), and (**D**) reactome pathways enrichment of HTRA1 network following the default addition of 10 nodes to the generated network. (**E**) Interactome centered on cathepsin G (CTSG), and (**F**) reactome pathways enrichment of CTSG network. (**G**) Interactome centered on alpha-1-acid-glycoprotein 1 (AGP1), and (**H**) reactome pathways enrichment of AGP1 network. In the STRING networks, nodes represent proteins and edges represent known or predicted protein–protein associations. Edge colors denote evidence types: teal blue, curated database annotations; pink, experimental evidence; green, gene neighborhood; red, gene fusions; blue, gene co-occurrence; yellow, text mining; black, co-expression; and light blue, protein homology. Interactions were visualized using a medium confidence score (0.4) and a false discovery rate (FDR) threshold of 5%. Reactome pathway enrichment was performed using the STRING platform with the druggable genome (6,805 genes) as the background set. Additional parameters included signal ≥ 0.01, strength ≥ 0.01, and FDR ≤ 0.05, with enriched pathways grouped at a similarity threshold ≥ 0.7. FDR represents *p*-values adjusted using the Benjamini-Hochberg correction method to correct for multiple comparisons

STRING database retrieved known and predicted PPIs based on computational predictions, experimental data, co-expression data, and text mining. Initially, to understand the collective role of differentially abundant proteins and their interactions, all eight differentially abundant proteins were input into the STRING database, generating a network. The collective interactome was enhanced by the default addition of 5 more nodes to the generated network to obtain the final graph with a PPI enrichment *p*-value of 7.93e-13. Enhancement indicated that the input proteins were at least partially biologically connected as a group. Next, individual proteins upregulated in OADM were input and analyzed for associations. Networks with PPI enrichment were obtained for HTRA1 (*p*-value = 0.305), CTSG (*p*-value = 1.02e-10), and AGP1 (*p*-value = 4.67e-11). Functions of the interacting proteins were deduced from the literature and UniProt database (Additional Table 2). By and large, all the interactomes contribute towards understanding the plausible associations behind the OADM disease condition. Reactome pathways enrichment was additionally performed to infer the functional roles of the proteins along with the biological significance of the network (Fig. 7). The details are discussed in Additional Table 3.

**Table 2:** ELISA-based validation and exploratory ROC performance of key OADM-associated proteins in SF and serum from OA and OADM patients. Data evaluated using the non-parametric tests: ^#^Mann-Whitney test, and ^$^Mann-Whitney *U* test. *Bias-corrected and accelerated (BC_a_) 95% confidence intervals (CIs), estimated using bootstrap resampling (1000 iterations; random number seed: 978). ^##^Exploratory ROC-derived threshold associated with OADM status (validation cohort; *n* = 24). *p* < 0.05 represents statistically significant data

Protein name		HtrA serine protease 1 (HTRA1)		Cathepsin G (CTSG)		Alpha-1-acid glycoprotein 1 (AGP1)	
Sample type		SF	Serum	SF	Serum	SF	Serum
**Fold change (OADM/ OA)**		2.7-fold	2.3-fold	1.5-fold	4.3-fold	1.6-fold	1.9-fold
***p*** **-value** ^**#**^		0.0001	0.0239	0.0371	0.0002	0.0001	0.0163
**AUC analysis**	**AUC (%)**	82.5%	69.1%	67.6%	81.5%	82.1%	70.3%
	***p*** **-value (of AUC)** ^**$**^	< 0.0001	0.018	0.028	< 0.0001	< 0.0001	0.01
	95% CI (%)*	65.8–92.9	52.4–83.2	50.2–81.1	62.9–91.7	65.8–92.2	52.5–83.4
**Youden index** ***J***	**Youden index** ***J***	0.58	0.46	0.38	0.58	0.67	0.50
	95% CI (of Youden index)*	0.33–0.75	0.21–0.63	0.17–0.54	0.33–0.75	0.46–0.83	0.25–0.71
	**Criterion** ^**##**^	> 0.82 ng/mL	> 0.62 ng/mL	> 2.48 ng/mL	> 0.82 ng/mL	> 4714 µg/mL	> 8408 µg/mL
	95% CI (of criterion)*	> 0.5 to > 1.26	> 0.56 to > 1.34	> 1.76 to > 3.6	> 0.79 to > 0.82	> 4652 to > 8802	> 8146 to > 18,762
	**Sensitivity (%)**	75%	79.2%	50%	62.5%	95.8%	75%
	**Specificity (%)**	83.3%	66.7%	87.5%	95.8%	70.8%	75%

### ELISA-based verification and validation of key OADM-associated proteins and assessment of translational relevance by SF-serum correlation and exploratory ROC analyses

#### Verification of SWATH-MS-identified proteins by ELISA

The three most differentially abundant proteins identified by SWATH-MS, HTRA1, CTSG, and AGP1, were first verified by ELISA in SF and paired serum samples from the discovery cohort (*n* = 6 per group). Consistent with the proteomic findings, all three proteins exhibited significantly higher abundance in OADM compared with OA. Specifically, SF levels of HTRA1, CTSG, and AGP1 were increased by 2.5-fold (*p* = 0.0002), 2.2-fold (*p* = 0.0006), and 1.7-fold (*p* < 0.0001), respectively, in OADM patients relative to OA. Corresponding increases were also observed in paired serum samples, with fold changes of 2.2 (*p* = 0.0050) for HTRA1, 1.6 (*p* = 0.0070) for CTSG, and 2.0 for AGP1 (*p* = 0.0140). These results confirmed the reproducibility of the differential abundance patterns observed in the discovery-phase proteomic analysis.

#### Validation of candidate proteins in an expanded cohort

**Fig. 8:**
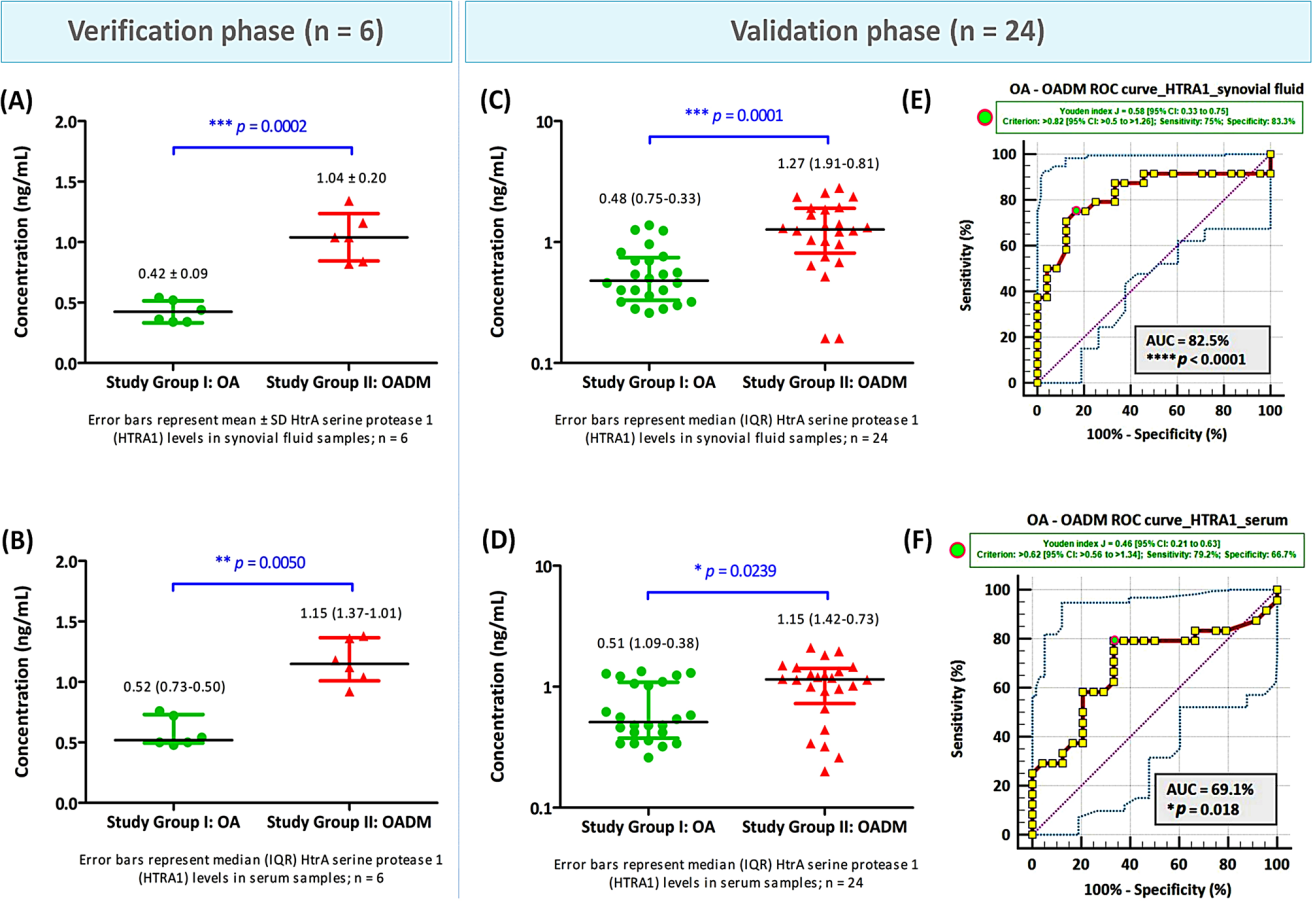
Quantification of HtrA serine protease 1 (HTRA1) levels in SF and serum from OA and OADM patients. HTRA1 concentrations (ng/mL) were measured by ELISA in (**A**) SF and (**B**) paired serum samples from the verification cohort (*n* = 6 per group), and in (**C**) SF and (**D**) paired serum samples from the independent validation cohort (*n* = 24 per group). Data normality was assessed using the Shapiro-Wilk and the Lilliefors-corrected Kolmogorov-Smirnov tests. Normally distributed data were analyzed using a two-tailed Welch’s t-test, whereas skewed or mixed-distribution data were analyzed using the non-parametric Mann-Whitney test. Results are presented as Mean ± SD for normal data and Median (IQR) for non-normal data. ROC curve analyses evaluating the ability of HTRA1 levels to discriminate between OA and OADM were performed using ELISA-derived concentrations in (**E**) SF and (**F**) serum from the validation cohort (*n* = 24). The AUC values with *p* < 0.05 indicate statistically significant discriminatory performance between the two study groups. The 95% CIs were generated using BC_a_ bootstrapping (1000 iterations; random number seed = 978). *p < 0.05, **p < 0.01, ***p < 0.001, and ****p < 0.0001 represent statistically significant data

**Fig. 9:**
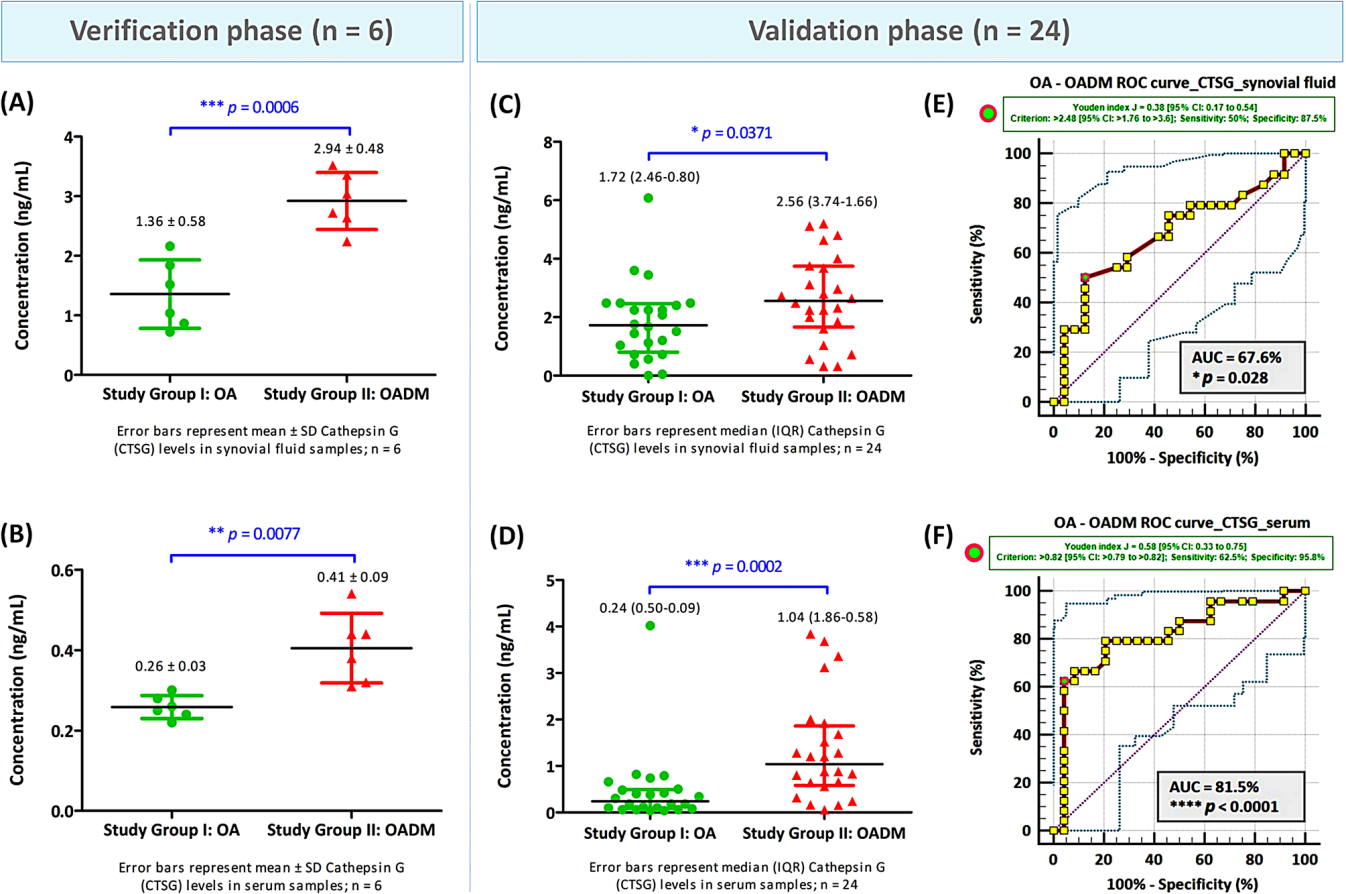
Cathepsin G (CTSG) concentration (ng/mL) in SF and serum collected from patients enrolled in different study groups. ELISA was performed to quantify CTSG levels in (**A**) SF and (**B**) paired serum samples from patients included in the verification phase of the study (*n* = 6), and in (**C**) SF and (**D**) paired serum samples from patients included in the validation phase (*n* = 24). Normality of data distribution was evaluated using both the Shapiro-Wilk and the Lilliefors-corrected Kolmogorov-Smirnov tests. Based on these assessments, parametric two-tailed Welch’s t-tests were applied to normally distributed variables, while non-parametric Mann-Whitney tests were used for skewed or mixed-distribution datasets. Data are reported as Mean ± SD for normally distributed variables and as Median (IQR) for non-normally distributed variables. Panels (**E**) and (**F**) depict OA–OADM ROC curves for CTSG concentrations in SF and paired serum samples, respectively, as determined by ELISA (*n* = 24). The significance of the AUC values for both ROC analyses (*p* < 0.05) indicates that CTSG levels in both SF and serum effectively discriminate between OA and OADM groups. The 95% CIs were generated using BC_a_ bootstrapping (1000 iterations; random number seed = 978). *p < 0.05, **p < 0.01, ***p < 0.001, and ****p < 0.0001 represent statistically significant data

**Fig. 10:**
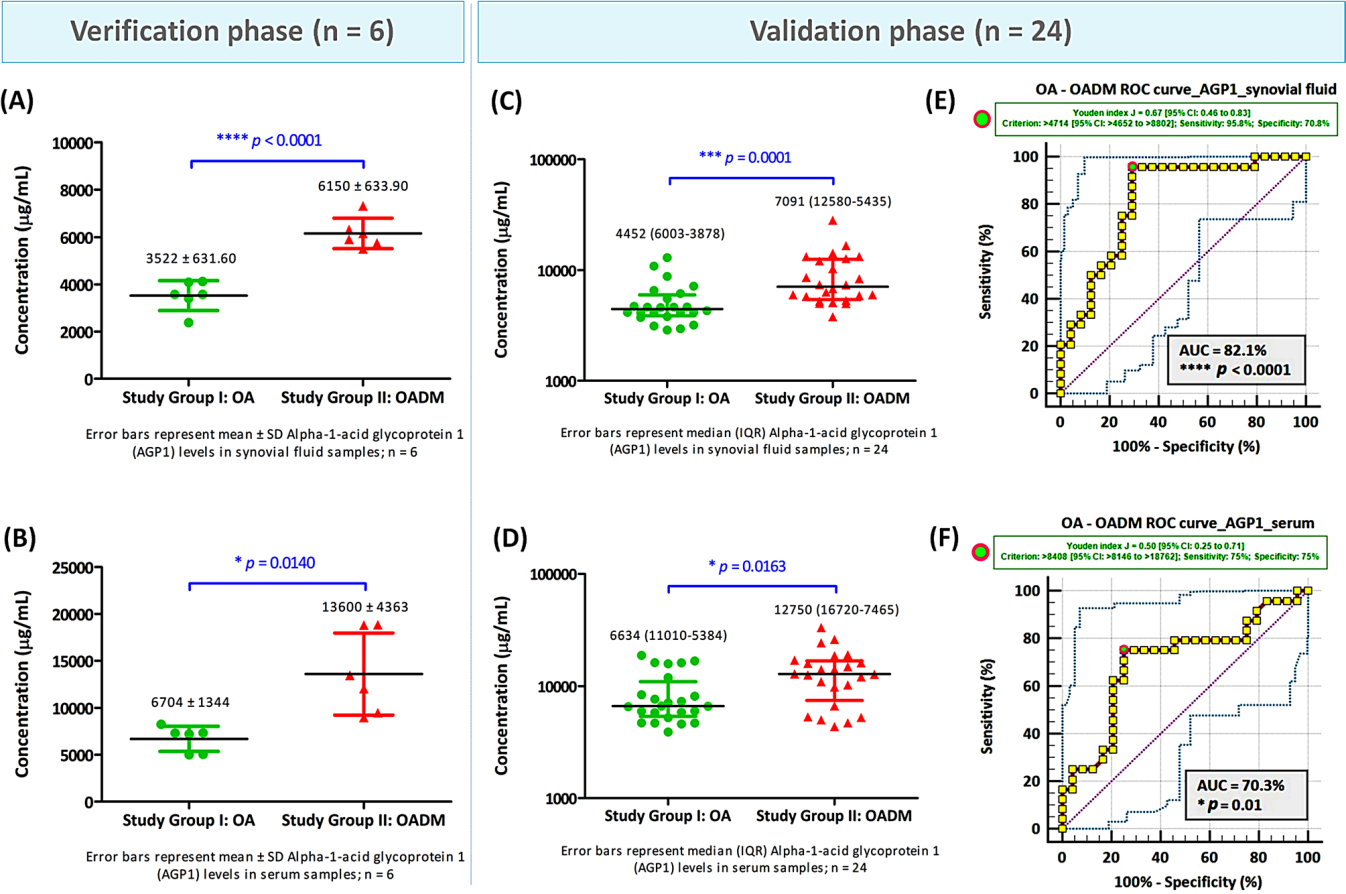
Differential abundance of alpha-1-acid glycoprotein 1 (AGP1) in SF and serum of OA and OADM patients. AGP1 concentrations (µg/mL) were quantified by ELISA in synovial fluid (SF) and paired serum samples obtained from patients across different study groups. Data distribution was tested for normality using the Shapiro-Wilk and the Lilliefors-corrected Kolmogorov-Smirnov tests. Welch’s two-tailed t-test was used for normally distributed data, and the Mann-Whitney test was applied to skewed or mixed distributions. Values are expressed as Mean ± SD or Median (IQR), as appropriate. Panels (**A**) and (**B**) show AGP1 levels in SF and serum, respectively, from the verification cohort (*n* = 6), while panels (**C**) and (**D**) depict AGP1 concentrations measured in SF and serum from the independent validation cohort (*n* = 24). AGP1 levels were consistently elevated in OADM compared with OA in both biological fluids. Panels (**E**) and (**F**) represent ROC curves assessing the discriminative performance of AGP1 concentrations in SF and serum, respectively, for differentiating OADM from OA. Statistically significant AUC values (*p* < 0.05) indicate diagnostic relevance. The 95% CIs were generated using BC_a_ bootstrapping (1000 iterations; random number seed = 978). *p < 0.05, ***p < 0.001, and ****p < 0.0001 denote statistically significant data

The differential abundance of HTRA1, CTSG, and AGP1 was subsequently evaluated in an expanded validation cohort comprising paired SF and serum samples from 24 OA and 24 OADM patients. In this expanded cohort, all three proteins remained significantly elevated in both SF and serum from OADM patients compared with OA, confirming the robustness of the observed associations across cohorts and biological matrices. HTRA1 exhibited a 2.7-fold (*p* = 0.0001) increase in SF and a 2.3-fold (*p* = 0.0239) increase in serum in OADM compared with OA (Fig. 8). CTSG levels displayed a 1.5-fold (*p* = 0.0371) increase in SF and a 4.3-fold (*p* = 0.0002) increase in serum in OADM compared with OA (Fig. 9), while AGP1 showed 1.6-fold (*p* = 0.0001) and 1.9-fold (*p* = 0.0163) increases in SF and serum in OADM compared with OA (Fig. 10). ELISA-based quantification demonstrated consistent directional changes for all three candidates, supporting their association with OADM-related pathophysiology and reinforcing their relevance beyond the discovery and verification phases.

#### Correlation between SF and serum levels of validated proteins in OADM patients

**Fig. 11:**
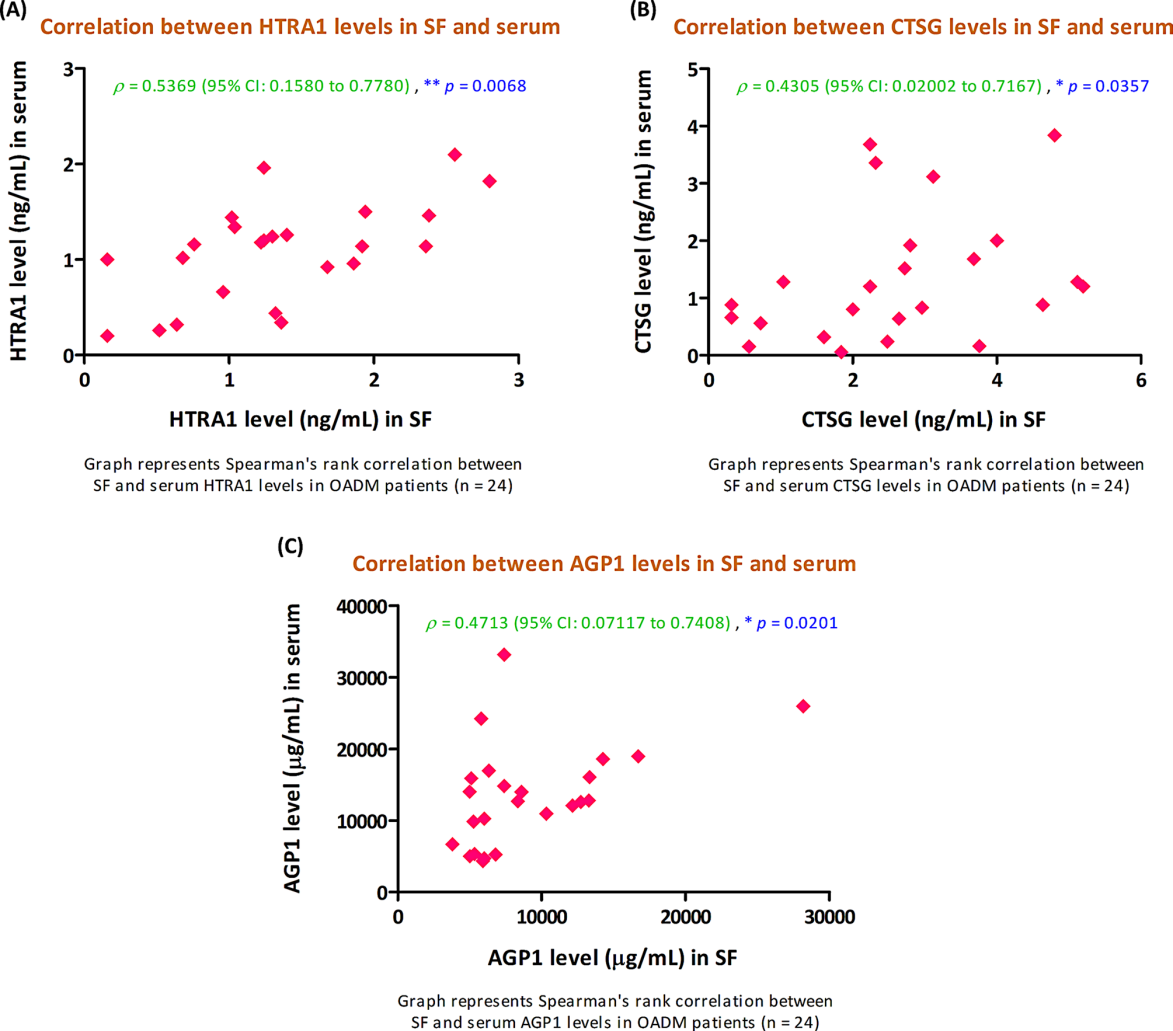
Association between SF and circulating levels of HTRA1, CTSG, and AGP1 in OADM patients. Scatter plots depict the correlation between SF and paired serum concentrations of (**A**) HTRA1, (**B**) CTSG, and (**C**) AGP1, measured by ELISA, in the OADM validation cohort (*n* = 24). Paired SF and serum levels for each subject are shown as individual data points. Spearman’s rank correlation analysis demonstrated statistically significant positive associations between SF and serum levels for all three proteins, indicating concordant abundance patterns across local joint and systemic compartments in OADM. Spearman’s rank correlation coefficient (*ρ*) and corresponding two-tailed *p*-values are shown for each panel. Correlation analyses were exploratory and not adjusted for multiple comparisons

Exploratory Spearman correlation analyses demonstrated statistically significant positive associations between SF and serum concentrations of all three validated proteins within the OADM validation cohort (*n* = 24). HTRA1 concentrations in SF showed a significant positive correlation with corresponding serum levels [Spearman’s rank correlation coefficient (*ρ*) = 0.5369 (95% CI: 0.1580 to 0.7780), *p* = 0.0068]. Similarly, CTSG and AGP1 concentrations in SF were positively correlated with their paired serum levels [CTSG: *ρ* = 0.4305 (95% CI: 0.02002 to 0.7167), *p* = 0.0357; AGP1: *ρ* = 0.4713 (95% CI: 0.07117 to 0.7408), *p* = 0.0201] (Fig. 11). These findings indicate concordant abundance patterns of these proteins across local joint and systemic compartments in OADM patients, supporting their potential translational relevance as circulating biomarkers reflective of joint-associated pathological processes.

#### Exploratory ROC analysis of validated proteins

Exploratory ROC analyses were conducted in the validation cohort to evaluate the discriminatory performance of HTRA1, CTSG, and AGP1 in distinguishing OADM from OA (Table 2). To account for bias and uncertainty associated with the limited sample size, AUC estimates were accompanied by BC_a_ 95% CIs derived from bootstrap resampling. Exploratory ROC analysis of HTRA1 showed AUC values of 82.5% (SF) and 69.1% (serum). Exploratory cut-off levels of > 0.82 ng/mL (SF) and > 0.62 ng/mL (serum) were associated with OADM status within the studied cohort (Fig. 8). Exploratory ROC analysis of CTSG yielded AUC values of 67.6% for SF and 81.5% for serum. Within the studied cohort, CTSG concentrations exceeding 2.48 ng/mL in SF, and 0.82 ng/mL in serum were associated with OADM status (Fig. 9). Exploratory ROC analysis of AGP1 revealed AUCs of 82.1% (SF) and 70.3% (serum), and exploratory cut-off values of > 4714 µg/mL (SF) and > 8408 µg/mL (serum) (Fig. 10). As cut-off values were derived and evaluated within the same cohort, these ROC analyses are interpreted as exploratory and cohort-specific. Nevertheless, the observed discriminatory trends support the potential relevance of these proteins as molecular correlates of OADM.

Collectively, ELISA-based verification, expanded validation, concordant SF-serum correlations, and exploratory ROC analyses indicate that HTRA1, CTSG, and AGP1 are reproducibly elevated in OADM and exhibit coherent behavior across local and systemic compartments. While these findings support their relevance as candidate biomarkers associated with metabolic and inflammatory alterations in OADM, further functional investigations and validation in larger, independent cohorts will be required to establish their mechanistic roles and clinical utility.

## Discussion

Lack of understanding regarding OADM pathology warranted a deeper exploration of the underlying molecular dynamics, besides emphasizing the need for diagnostic tools and therapies that can diagnose and monitor OA and T2DM disease conditions concurrently. The present study was, hence, conducted with the aim of identifying glycated/ glycosylated protein biomarkers and potential therapeutic agents for OA management in patients with T2DM.

Demographic and laboratory assessment showed a higher prevalence of OA and OADM among North Indian females around 65 years of age, indicating potential age- and sex-related influences [4, 22]. Increased disease prevalence was also observed in overweight individuals of both sexes, consistent with prior studies [4]. The similarity in age, sex, BMI, and KL grade between OA and OADM groups suggests that differences in disease severity are unlikely to be driven solely by general clinical characteristics, but may instead reflect diabetes-related metabolic factors, including hyperglycemia-induced protein modification and the accumulation of AGEs within the joint microenvironment [10]. The absence of significant differences in total protein concentrations in SF and serum further supports the notion that qualitative proteomic alterations, rather than global protein abundance, underlie the observed differences.

To improve clinical characterization, information on key comorbidities, specifically hypertension and hypothyroidism, was included in the analysis. Hypertension was more prevalent in patients with OADM, particularly in the validation cohort, whereas hypothyroidism was observed in a smaller proportion of patients in both groups. Although these comorbidities may contribute to systemic inflammation and metabolic stress, their distribution does not fully explain the observed proteomic differences between OA and OADM. Detailed data on diabetes duration and medication use were not uniformly available and are acknowledged as potential contributors to inter-individual variability in proteomic profiles. To partially address this limitation, available metabolic indicators, including FBS and HbA1c, were used as surrogate indicators of glycemic burden and metabolic status. Collectively, these findings underscore the importance of considering metabolic control and diabetes-related systemic factors when investigating OA pathogenesis in patients with T2DM, and they support the need for future longitudinal studies with more comprehensive clinical characterization to delineate the relative contributions of glycemic duration, comorbidities, and pharmacotherapy. Such efforts will be essential for integrating metabolic management into therapeutic strategies aimed at mitigating joint degeneration in OADM.

For the discovery phase analysis, SF samples were processed through a PBA-based glycoprotein enrichment kit. pH-dependent attachment of the OH group of boronic acids to the 1, 2-cis-diol groups of biomolecules, and the selective presence of these 1, 2-cis-diol groups in glycated/ glycosylated proteins [23], formed the basis for the usage of PBA-based columns. Moreover, the kit aimed to remove > 90% of the non-glycosylated fraction of proteins constituted by albumin. Removal of albumin was necessary since enrichment of serum glycated proteins by boronic acid affinity chromatography column, followed by MS analysis of the enzymatic digest of the enriched fraction, has been shown to yield numerous non-glycated peptides that were mostly from serum albumin, and were a result of non-specific binding of serum proteins to PBA-agarose packed chromatography column [24]. Moreover, albumin also acts as a principal target for glycation because of its high abundance and the presence of a large number of Arg and Lys residues [25], masking the presence of low abundance proteins, which might be important players behind OADM disease pathogenesis.

Next, the enriched samples were analyzed by LC-MS/MS to enable quantitative profiling of cis-diol-containing glycated/ glycosylated proteomic alterations associated with OA and OADM. Quantification was performed using DIA-based SWATH-MS, with peptide identification supported by a well-established, SF-specific spectral library, consistent with standard DIA proteomic workflows. In line with prior systematic evaluations, this approach prioritizes spectral library quality and biological relevance to achieve reproducible and accurate relative quantification across biological conditions [26]. Accordingly, the analytical strategy employed here enabled reliable comparative assessment of joint-relevant protein signatures between OA and OADM groups, and the resulting data are interpreted as biologically meaningful and confidently quantifiable SF proteomic features.

SWATH-MS profiling revealed substantial proteomic diversity between the two disease conditions. Following rigorous data normalization and statistical analysis, several cis-diol-containing glycation/ glycosylation-enriched proteins were identified as differentially abundant in OADM relative to OA. Among these, HTRA1, CTSG, and AGP1 emerged as the most prominently upregulated proteins, whereas PFN1, GPLD1, PROS1, APOA2, and APOA4 were among the most significantly downregulated.

Concerning the upregulated proteins, HTRA1 is an ECM-degrading serine protease, previously reported to be overexpressed in cartilage affected by OA [27], as well as both OA and T2DM [28]. It is a major determinant that accelerates the proteolytic activity of matrix metalloproteinases (MMPs), which drive cartilage destruction by cleaving prime structural components, such as fibronectin and aggrecan [29, 30]. The observed upregulation of HTRA1 by Murayama et al. was associated with hallmarks of OA progression, including elevated joint inflammation and cellular senescence [31]. Diabetes-mediated pro-inflammatory environment, characterized by hyperglycemia and the generation of AGEs [32], may further stimulate HTRA1 levels, as is evident from increased HTRA1 levels in gestational diabetes mellitus [33] and proliferative diabetic retinopathy [34]. Existing literature thus supports the upregulation of this protein in the co-morbid OADM condition and indicates a potential association with pathways involved in cartilage degradation and inflammation.

Another protein, CTSG, is also a serine protease, well documented for its role in inflammation, immune regulation, and tissue remodeling [35]. It contributes to ECM breakdown owing to its role in the degradation of proteoglycans and collagen [36]. The protein has been shown to be induced in SF of OA patients while possessing a prominent ability to degrade lubricin, a glycoprotein present in SF that is crucial for joint lubrication and cartilage protection [37]. CTSG was also identified as a target for the progression of rheumatoid arthritis owing to its involvement in the degradation of collagen and the ECM [38]. Accordingly, elevated CTSG levels are indicative of an association with increased joint erosion and inflammatory activity. Previous studies have reported increased CTSG expression under conditions of hyperglycemia and systemic inflammation [39], suggesting a potential link to impaired joint homeostasis in OADM. In the present study, the observed upregulation of CTSG is therefore interpreted as a reflection of heightened systemic inflammatory burden associated with OADM co-morbidity.

The increased abundance of AGP1, an acute-phase glycoprotein that modulates the host immune responses and inflammation, reflects the characteristic T2DM-related outcomes, including pronounced systemic inflammation and an altered metabolic status. An earlier study revealed that AGP1 interacts with immune cells, amplifies the production of cytokines, and contributes to synovitis and cartilage damage [17]. Elevated AGP1 levels have also been documented in inflammatory conditions including rheumatoid arthritis [40] and in patients with T2DM [41]. Consistent with the present findings, a systematic review by Le et al. highlighted that AGP1 is elevated in individuals with T2DM due to the prevalence of low-grade inflammation [42]. Collectively, these observations suggest that elevated AGP1 in OADM may be associated with enhanced inflammatory signaling within the joint microenvironment, potentially contributing to cartilage degeneration and pain.

About the downregulated proteins, profilin-1 is a cytoskeletal remodeling protein essential for cellular functions, encompassing migration and adhesion [43]. Reduced PFN1 levels in diabetic conditions have been linked to excessive deposition of AGEs due to hyperglycemia, which affects cytoskeleton proteins, subsequently damaging the actin cytoskeleton [44]. Experimental silencing of PFN1 has been shown to impair actin dynamics [43], suggesting a potential impact on cellular structural integrity, including that of chondrocytes. PFN1 downregulation in OA, as revealed from SWATH-MS analysis of SF [14], may reflect impaired chondrocyte survival, decreased chondrocyte activity, and impacted cartilage maintenance. These observations support an association between reduced PFN1 levels and dysregulated cytoskeletal and ECM homeostasis in both OA and diabetes, indicating that its downregulation in OADM may reflect disease-associated cellular stress and matrix remodeling rather than a direct causal driver of cartilage degradation.

GPLD1 is an enzyme that cleaves GPI-anchored proteins from the cell membrane and has been implicated in lipid metabolism and modulation of inflammatory processes [45]. Although its direct involvement in OA or T2DM has not been well defined, the reduced levels of GPLD1 observed in OADM may indicate disrupted lipid homeostasis and altered inflammatory regulation. These changes may be associated with altered synovial and cartilage tissue homeostasis in the presence of metabolic dysregulation.

PROS1, a vitamin K-dependent glycoprotein and an anticoagulant factor [46], functions as a bone matrix component synthesized and secreted by osteoblasts. It plays a role in bone turnover and bone mass regulation [47]. Vitamin K plays a role in OA pathogenesis through effects on bone and cartilage proteins, and its deficiency was shown to be related to the prevalence and progression of OA [48]. Given that the balance between bone formation and resorption is disrupted in OA, the reduced abundance of the vitamin K–dependent protein PROS1 observed in the present study may reflect altered bone remodeling and compromised bone–cartilage homeostasis in the OADM condition, potentially associating it with increased joint degeneration.

Apolipoproteins are well-known integral components of high-density lipoproteins (HDLs) that play crucial roles in transporting lipids, regulating inflammation, and providing defense against oxidative stress [49]. HDL dysfunction has been profoundly shown to correlate with increased chronic inflammation and oxidative stress in joint tissues in OA, which may aggravate cartilage degradation and incur changes in the subchondral bone [50]. Reports show that low levels of apolipoproteins, APOA2 and APOA4, are linked to an increased risk of diabetes [51, 52]. Downregulated levels of these anti-inflammatory apolipoproteins in the OADM condition may reflect altered lipid metabolism and compromised HDL functionality, which could be associated with a pro-inflammatory joint environment and impaired tissue repair.

Collectively, the differential abundance of PBA-enriched cis-diol-containing glycation/ glycosylation-associated proteins observed in OADM suggests potential molecular links between diabetes-associated features, such as chronic hyperglycemia and oxidative stress, and heightened inflammatory responses, joint degeneration, and impaired tissue repair. The findings underscore the relevance of cis-diol–associated protein modifications in SF to OADM pathology. In this context, such modifications may be associated with altered abundance and/ or functional regulation of proteins such as HTRA1, CTSG, and AGP1, which have been implicated in inflammatory and degradative processes within the joint.

The set of abundant proteins obtained was further subjected to correlation analysis. Strong correlations between the proteins provided critical insights into the molecular engagement between OA and OADM disease conditions, and insights into their biological relatedness. The analysis revealed that proteins with similar expression patterns cluster and are potentially co-regulated. Pearson’s correlation coefficient ‘*r*’ analysis presented both a positive and negative linear relationship among the key glycated/ glycosylated proteins, suggestive of coordinated expression patterns and functional associations. These associations were, however, not indicative of causal relationships and require further experimental investigation to dissect the mechanisms underlying OADM pathogenesis. Positive correlations, as observed between apolipoproteins APOA4 and APOA2, PROS1 and PFN1, APOA2 and GPLD1, and APOA4 and GPLD1, suggest that these proteins may be coordinately regulated in OADM. Conversely, negative correlations observed between AGP1 and APOA4, and between HTRA1 and PFN1, may reflect reciprocal or compensatory regulatory patterns, in which changes in the abundance of one protein are associated with opposing changes in another. The correlations imply that the proteins upregulated or downregulated in the OADM condition are not independent players, but rather form part of an interconnected molecular network associated with disease-related processes.

Analysis of differentially abundant proteins by unsupervised multivariate approaches, such as PCA and HCA, revealed clear proteomic distinctions between OA and OADM groups. Pathway enrichment analyses further provided contextual insight into biological processes potentially associated with OADM co-pathogenesis. PCA demonstrated statistically significant group separation, supported by PERMANOVA (R^2^ = 0.64), indicating that a substantial proportion of variance in the dataset is attributable to differences between the two clinical conditions. The distinct clustering patterns and limited overlap of group confidence ellipses suggest systemic alterations in the SF proteome associated with OADM. Importantly, because PCA was performed without adjustment for covariates, such as glycemic markers, inflammatory indices, or lipid profile-related variables, these differences are interpreted as reflecting broader metabolic and inflammatory dysregulation related to OADM, rather than exhibiting biomarker specificity or showing joint-exclusive molecular effects.

In the context of the hyperglycemic milieu characteristic of OADM, diabetes-associated metabolic stress, including protein glycation/ glycosylation and AGE-related processes, has been shown to influence protein abundance and expression patterns [8]. Consistent with prior reports linking hyperglycemia and AGEs to altered gene and protein expression and enhanced inflammation in OA patients with diabetes [53, 54], the observed proteomic separation likely reflects the combined influence of systemic metabolic dysregulation and inflammatory signaling on the SF proteome.

Heatmap-based hierarchical clustering further supported these findings by delineating coordinated abundance patterns among upregulated and downregulated protein subsets. In particular, HTRA1, CTSG, and AGP1 clustered as upregulated proteins in OADM and were identified as key PBA-enriched cis-diol-containing proteomic signatures associated with the OADM metabolic phenotype. Collectively, unsupervised multivariate analyses patterns suggest that cis-diol-associated glycation/ glycosylation modifications may be linked to inflammatory and degradative processes within the joint, while also underscoring the contribution of T2DM-related systemic metabolic alterations to OA pathogenesis, in line with the concept of metabolic OA [6]. However, future studies incorporating multivariable modeling and stratification by metabolic and inflammatory markers will be required to disentangle systemic versus joint-local molecular changes.

Next, observations from PCA and HCA motivated further exploration of biological pathways potentially associated with the co-pathogenic OADM condition. Accordingly, GO and KEGG bioinformatics analyses were performed on the significantly upregulated proteins in OADM. GO analysis revealed a predominant association of upregulated proteins with ECM components and processes. Cellular component enrichment in collagen-containing ECM suggests an association with matrix remodeling, a recognized feature of OA pathology [55]. Enrichment in biological processes such as ECM disassembly and organization, angiotensin regulation, and neutrophil degranulation, point toward coordinated alterations in matrix structure and inflammatory signaling in OADM [55–57]. Molecular function analysis revealed enrichment in serine-type peptidase and hydrolase activities, which have been implicated in ECM turnover and inflammatory responses in joint disease [58, 59]. KEGG pathway analysis revealed involvement in RAS dysregulation, consistent with prior reports linking RAS signaling to synovial inflammation, OA progression, and T2DM-related metabolic disturbances [60, 61]. Overall, these observations are consistent with existing literature associating elevated angiotensin levels with cartilage degradation and systemic manifestations such as arterial hypertension and insulin resistance [7, 61]. Importantly, the pathway enrichment patterns observed here reflect associative molecular signatures and do not establish direct causal mechanisms, suggesting that systemic metabolic and inflammatory alterations in T2DM may influence joint pathology in OADM.

GO analysis of the downregulated proteins provided insight into the molecular alterations potentially associated with the OADM condition. A substantial proportion of these proteins were enriched in the cellular component of blood microparticles, suggesting possible alterations in intercellular communication and transport processes [62], which are relevant to both joint and systemic metabolic homeostasis. Enrichment of biological processes, including lipid catabolism, triglyceride metabolism, and glycerolipid catabolism, indicates attenuation of lipid degradation pathways, which may be associated with lipid accumulation and disturbed metabolic balance commonly reported in OA and T2DM [63]. In addition, molecular function analysis revealed enrichment in sterol transfer activity, pointing toward altered cholesterol handling and perturbed lipid homeostasis in OADM [64]. The enrichment of KEGG pathways, such as cholesterol metabolism, GPI-anchor biosynthesis, fat digestion and absorption, PPAR signaling, and complement and coagulation cascades, further suggested a multifaceted disruption in metabolic and inflammatory pathways in the co-pathogenic condition. Dysregulation of cholesterol metabolism and GPI-anchor biosynthesis may influence cell membrane composition and signaling, with potential implications for chondrocyte function and joint tissue integrity in OA [65]. Similarly, reduced activity in fat digestion and absorption pathways and attenuated PPAR signaling have been associated with lipid dysregulation and insulin resistance, factors that may indirectly influence joint pathology in OADM [66, 67]. Enrichment of complement and coagulation cascades further suggests altered immune and inflammatory responses [68, 69], which are frequently observed in OA and T2DM.

Taken together, GO and KEGG analyses of downregulated proteins point to a complex molecular landscape in OADM, characterized by altered lipid metabolism, immune regulation, and cellular communication. These findings emphasize the systemic nature of OADM, wherein metabolic dysregulation may be reflected in local joint-associated proteomic changes. Rather than indicating discrete disease-driving mechanisms, these enrichments point to biological processes that warrant further investigation to clarify their contribution to OA progression in the context of diabetes.

Complementary to pathway enrichment, STRING-derived protein–protein interaction networks and Reactome analysis revealed coordinated associations among differentially abundant proteins, highlighting the interconnected nature of inflammatory, metabolic, and ECM-related processes in OADM. This network-based perspective provides additional context for understanding how multiple pathways may converge in the co-occurrence of OA and T2DM and offers a framework for prioritizing targets for future functional studies.

The last phase of the study evaluated selected proteomic findings in both SF and serum samples from an expanded cohort, showing that proteins identified as upregulated in the OADM SF by SWATH-MS are also detectable at elevated levels systemically. These observations suggest that the identified proteins may be associated with both local joint pathology and broader metabolic or inflammatory alterations characteristic of the OADM condition. Building on these validation findings, exploratory correlation analyses demonstrated significant positive associations between SF and serum concentrations of HTRA1, CTSG, and AGP1 within the OADM validation cohort. The observed SF-serum concordance supports the presence of coordinated abundance patterns across local and systemic compartments, implying that elevated circulating levels of these proteins may, at least in part, reflect joint-associated pathological processes rather than representing purely systemic or incidental changes. From a translational perspective, such coupling between SF and serum is particularly relevant, as it supports the feasibility of using minimally invasive serum-based measurements to capture disease-associated molecular signatures linked to joint degeneration in OADM. While these correlations are exploratory and do not establish causality or directionality, they enhance the biological plausibility of these proteins as integrative markers of OADM-related inflammatory and metabolic dysregulation.

Finally, the translational potential of these candidates was further explored using ROC curve analyses, which suggested that HTRA1, CTSG, and AGP1 exhibit discriminatory capacity for distinguishing OADM from OA within the studied cohort. Although the observed AUC values indicate moderate to good classification performance in both SF and serum, these analyses remain exploratory, as cut-off values were derived and evaluated within the same validation cohort. The modest sample sizes in both the discovery and validation phases further limit statistical power and generalizability. Consequently, these proteins can be best regarded as candidate biomarkers associated with OADM-related pathophysiology. Independent validation in larger, multi-center cohorts, coupled with formal cross-validation strategies, will be essential to establish their clinical utility as diagnostic markers and further mechanistic studies are needed to classify these as confirmed drivers of disease processes. Nevertheless, the consistent elevation of these proteins across matrices, their SF-serum concordance, and their association with inflammatory and metabolic pathways collectively support their relevance to the inflammatory milieu commonly observed in OADM and provide a strong rationale for future functional and mechanistic investigations.

## Conclusions and future perspectives

The present study identifies HTRA1, CTSG, and AGP1 as PBA-enriched cis-diol-containing glycated/ glycosylated protein signatures that are differentially abundant in OA patients with T2DM, highlighting their potential relevance as OADM-associated candidate biomarkers. The findings reflect proteomic changes in OADM, encompassing both glycated and glycosylated proteins, and provide insight into molecular patterns linking metabolic dysregulation with joint pathology. The altered abundance of these proteins underscores the potential importance of post-translational modifications in OADM and suggests an association between systemic inflammation and disease severity in this co-morbid condition. Moreover, the observations are consistent with the concept that diabetes-associated metabolic stress, including hyperglycemia-related glycation/ glycosylation processes, may influence inflammatory and degradative pathways within the joint microenvironment.

Evaluation of these candidate proteins in both SF and serum supports their translational relevance and feasibility as potential biomarkers across biological matrices; however, the diagnostic performance observed in exploratory ROC analyses should be regarded as preliminary. Further studies in larger, independent, and multicenter cohorts are required to validate their diagnostic utility and broader clinical applicability. Importantly, future investigations incorporating glycation-site mapping or AGE-specific assays will be essential to delineate glycation-specific functional effects and to distinguish the relative contributions of glycation versus glycosylation. In addition, functional, longitudinal, and disease-relevant in vitro or in vivo models, including gain- and loss-of-function approaches and glycation-specific assays, will be required to clarify the biological roles of these proteins and to determine whether targeting glycation-related pathways, RAGE signaling, or the identified candidate proteins may have therapeutic relevance in OADM.

## Supplementary Information

Below is the link to the electronic supplementary material.

Supplementary Material 1

Supplementary Material 2

## Data Availability

All data generated or analyzed during this study are included in the manuscript (and its supplemental data). The MS proteomics data are available *via* the ProteomeXchange Consortium (PRIDE partner repository) with the dataset identifer PXD062955. Further information is available from the corresponding author on reasonable request.

## Abbreviations

ABC: Ammonium bicarbonate
ACN: Acetonitrile
AGEs: Advanced glycation end products
AGP1: Alpha-1-acid glycoprotein 1
AP: Anteroposterior
APOA: Apolipoprotein A-IV
APOA2: Apolipoprotein A-II
AUC-ROC: Area under the receiver operating characteristic curve
BCA: Bicinchoninic acid
BMI: Body mass index
BSA: Bovine serum albumin
CTSG: Cathepsin G
DIA: Data-independent acquisition
DTT: 1,4-dithiothreitol
ECM: Extracellular matrix
ELISA: Enzyme-linked immunosorbent assay
FBS: Fasting blood sugar
FDR: False discovery rate
GO: Gene Ontology
GPI: Glycosylphosphatidylinositol
GPLD1: Phosphatidylinositol-glycan-specific phospholipase D
HbA1c: Glycated hemoglobin
HCA: Hierarchical cluster analysis
HDLs: High-density lipoproteins
HTRA1: High-temperature requirement protein A1
IAA: Iodoacetic acid
IQR: Interquartile range
KEGG: Kyoto Encyclopedia of Genes and Genomes
KL: Kellgren-Lawrence
LC-MS/MS: Liquid chromatography-tandem mass spectrometry
ML: Machine learning
MMPs: Matrix metalloproteinases
OA: Osteoarthritis
OADM: Osteoarthritis and type 2 diabetes mellitus co-pathogenesis
OPD: Outpatient department
OT: Operation theater
PBA: Phenyl boronic acid
PCA: Principal component analysis
PERMANOVA: PERmutational Multivariate ANalysis of Variance
PFN1: Profilin-1
PPAR: Peroxisome proliferator-activated receptor
PPIs: Protein-protein interactions
PQPs: Peptide query parameters
PRIDE: PRoteomics IDEntification database
PROS1: Vitamin K-dependent protein S
RAGE: Receptors of advanced glycation end products
RAS: Renin-angiotensin system
SD: Standard deviation
SDS-PAGE: Sodium dodecyl sulphate-polyacrylamide gel electrophoresis
SF: Synovial fluid
STRING: Search Tool for the Retrieval of Interacting Genes/ Proteins
SWATH-MS: Sequential window acquisition of all theoretical mass spectra
T2DM: Type 2 diabetes mellitus
TAS: Total area sum
TKR: Total knee replacement
WHO: World Health Organization
