# Supplementary material for: PBA-enriched glycated/ glycosylated synovial fluid proteomic signatures associated with metabolic dysregulation and cartilage degeneration in osteoarthritis with type 2 diabetes
Source: J Transl Med. 2026 Mar 25;24:622. doi: 10.1186/s12967-026-07984-8 (PMC13137652; doi:10.1186/s12967-026-07984-8)
Supplement: Supplementary file 2 — Supplementary Material 2 [file 12967_2026_7984_MOESM2_ESM.docx]

**ADDITIONAL TABLE CAPTIONS**

1. **Table 1. List of 266 proteins obtained from analysis of SWATH-MS data, along with their abundances within different OA and OADM samples.** The raw protein abundances calculated from area-under-the-curve (AUC) values of peptide intensity peaks were normalized using total area sum (TAS) intensity method. The normalized abundances were further log2 transformed, followed by calculation of log2FC and FC values of the individual proteins. The data was statistically evaluated by a Student’s t-test, and filtered on the basis of *p*-value cut-off of < 0.05.
2. **Table 2. Protein interaction partners of (a) all the significant differentially abundant proteins in OADM, (b) high-temperature requirement protein A1 (HTRA1), (c) cathepsin G (CTSG), and (d) alpha-1-acid glycoprotein 1 (AGP1), with associated functional annotations.** The interaction study was performed using STRING v12.0 tool. Screening was done against *Homo sapiens* as the organism of interest, following FDR stringency of 0.05, while adjusting the confidence score to 0.4.
3. **Table 3. Biological significance of the pathways identified from Reactome database-based functional enrichment analysis of various interactomes generated using STRING v12.0, as deduced from the literature.**

**Table 2a. Protein interaction partners of all the significant differentially abundant proteins in OADM, with associated functional annotations.** The interaction study was performed using STRING v12.0 tool. Screening was done against *Homo sapiens* as the organism of interest, following FDR stringency of 0.05, while adjusting the confidence score to 0.4.

| **UniProt ID of the query protein** | **STRING protein** | % **identity/ interaction score** | **Annotated function according to literature and UniProt database** |
| --- | --- | --- | --- |
| A0A0N9Y7Q6 | Apolipoprotein A-IV (APOA4) | 100% | Apoa-IV is a major component of high-density lipoprotein (HDL). It belongs to the apolipoprotein A1/A4/E family. May also have a role in chylomicrons and very-low-density lipoprotein (VLDL) secretion and catabolism. |
| A0A117ID80 | Alpha-1-acid glycoprotein 1 (ORM1) | 100% | ORM belongs to the calycin superfamily of lipocalin family. It functions as transport protein in the blood stream. Binds various ligands in the interior of its beta-barrel domain. Also binds synthetic drugs and influences their distribution and availability in the body. Appears to function in modulating the activity of the immune system during the acute-phase reaction. |
| A0A100WT58 | Truncated apolipoprotein A-II (APOA2) | 100% | APOA2 belongs to the apolipoprotein A2 family. It may stabilize HDL structure by its association with lipids, and affect the HDL metabolism. |
| A0A117IFB2 | Phosphatidylinositol-glycan-specific phospholipase D (GPLD1) | 100% | GPLD1 hydrolyzes inositol phosphate linkage in proteins anchored by phosphatidylinositol glycans (GPI-anchor), thus releasing these proteins from the membrane. |
| A0A0N9XHA4 | Vitamin K-dependent protein S (PROS1) | 99.4% | It is an anticoagulant plasma protein, and a cofactor to activated protein C in degradation of coagulation factors Va and VIIIa. It helps to prevent coagulation and stimulates fibrinolysis. |
| A0A0N9XC43 | Profilin-1(PFN1) | 100% | PFN1 binds to actin and affects the structure of the cytoskeleton. At high concentrations, profilin prevents the polymerization of actin, whereas it enhances it at low concentrations. By binding to PIP2, it inhibits the formation of IP3 and DG. Inhibits androgen receptor (AR) and HTT aggregation; binding of G-actin is essential for its inhibition of AR. |
| A0A0N9XCR9 | Cathepsin G (CTSG) | 100% | It is a serine protease with trypsin- and chymotrypsin-like specificity. Cleaves complement C3. Has antibacterial activity against the Gram-negative bacterium *Pseudomonas aeruginosa*. The enzyme and antibacterial activity of CTSG is inhibited by lipopolysaccharide (LPS) from *P. aeruginosa*, Z-Gly-Leu-Phe-CH_2_Cl, and phenylmethylsulfonyl fluoride (PMSF). |
| A0A117IFN2 | Serine protease HTRA1 (HTRA1) | 100% | HTRA1 belongs to the peptidase S1C family. It is a serine protease with a variety of targets, including extracellular matrix (ECM) proteins, such as fibronectin. HTRA1-generated fibronectin fragments further induce synovial cells to up-regulate production of matrix metalloproteinases (MMPs), MMP1 and MMP3. May also degrade proteoglycans, such as aggrecan, decorin, and fibromodulin. Through cleavage of proteoglycans, may release soluble fibroblast growth factor (FGF)-glycosaminoglycan complexes that promote the range and intensity of FGF signals in the extracellular space. Regulates the availability of insulin-like growth factors (IGFs) by cleaving IGF-binding proteins. Inhibits signaling mediated by TGF-beta family members. This activity requires the integrity of the catalytic site, although it is unclear whether TGF-beta proteins are themselves degraded. By acting on TGF-beta signaling, may regulate many physiological processes, including retinal angiogenesis and neuronal survival and maturation during development. Intracellularly, degrades TSC2, leading to the activation of TSC2 downstream targets, including the mTOR pathway, which regulates cell growth and metabolism. The activation of mTOR, a key regulator of cell growth and metabolism, further results in the activation of downstream targets, including S6K, eEF2K, and AKT. |
| **Predicted functional partners** | | | |
|  | Heparin cofactor 2 (SERPIND1) | 0.801 | The thrombin inhibitor belongs to the serpin family. It is activated by the glycosaminoglycans, heparin, or dermatan sulfate. In the presence of the latter, HC-II becomes the predominant thrombin inhibitor in place of antithrombin III (AT-III). Also inhibits chymotrypsin , but in a glycosaminoglycan –independent manner |
|  | SAA2-SAA4 readthrough (SAA2-SAA4) | 0.689 | SAA2-SAA4 readthrough |
|  | Serum amyloid A-4 protein (SAA4) | 0.816 | SAA4 belongs to the SAA family and is a major acute phase reactant. It is a apolipoprotein of the HDL complex. |
|  | Apolipoprotein F (APOF) | 0.921 | APOF is a minor apolipoprotein that associates with LDL. It inhibits cholesteryl ester transfer protein (CETP) activity, and appears to be an important regulator of cholesterol transport. Also associates to a lesser degree with VLDL, Apo-AI, and Apo-AII. |
|  | Fibrinogen gamma chain (FGG) | 0.841 | FGG, together with fibrinogen alpha (FGA) and fibrinogen beta (FGB), polymerizes to form an insoluble fibrin matrix, a key component of blood clots and essential for hemostasis (stopping bleeding). In addition, functions during the early stages of wound repair to stabilize the lesion and guide cell migration during re-epithelialization. Was originally thought to be essential for platelet aggregation, based on *in vitro* studies using anti-coagulated blood. However, subsequent studies have shown that it is not absolutely required for thrombus formation *in vivo*. |

**Table 2b. Protein interaction partners of high-temperature requirement protein A1 (HTRA1), with associated functional annotations.** The interaction study was performed using STRING v12.0 tool. Screening was done against *Homo sapiens* as the organism of interest, following FDR stringency of 0.05, while adjusting the confidence score to 0.4.

| **Predicted functional partners of HTRA1 in STRING database** | **Interaction score** | **Annotated function according to literature and UniProt database** |
| --- | --- | --- |
| Aggrecan core protein 2 (ACAN) | 0.843 | This proteoglycan is a major component of ECM of cartilagenous tissues. A major function of this protein is to resist compression in cartilage. It binds avidly to hyaluronic acid *via* an N-terminal globular region. |
| E3 ubiquitin-protein ligase (XIAP) | 0.805 | It is a multi-functional protein which regulates not only caspases and apoptosis, but also modulates inflammatory signaling and immunity, copper homeostasis, mitogenic kinase signaling, cell proliferation, as well as cell invasion and metastasis. Acts as a direct caspase inhibitor. Directly binds to the active site pocket of CASP3 and CASP7 and obstructs substrate entry. Inactivates CASP9 by keeping it in a monomeric, inactive state. Acts as E3 ubiquitin-protein ligase regulating NF-kappa-B signaling and the target proteins, including RIPK1, CASP3, CASP7, CASP8, CASP9, MAP3K2/MEKK2, DIABLO/SMAC, AIFM1, CCS and BIRC5/survivin, for its E3 ubiquitin-protein ligase activity. Ubiquitinion of CCS leads to enhancement of its chaperone activity toward its physiologic target, SOD1, rather than proteasomal degradation. Ubiquitinion of MAP3K2/MEKK2 and AIFM1 does not lead to proteasomal degradation. Plays a role in copper homeostasis by ubiquitinating COMMD1 and promoting its proteasomal degradation. Can also function as E3 ubiquitin-protein ligase of the NEDD8 conjugation pathway, targeting effector caspases for neddylation and inactivation. Regulates the BMP signaling pathway and the SMAD and MAP3K7/TAK1 dependent pathways leading to NF-kappa-B and JNK activation. Acts as an important regulator of innate immune signaling *via* regulation of Nod-like receptors (NLRs). Protects cells from spontaneous formation of the ripoptosome, a large multi-protein complex that has the capability to kill cancer cells in a caspase-dependent and caspase-independent manner. Suppresses ripoptosome formation by ubiquitinating RIPK1 and CASP8. Acts as a positive regulator of Wnt signaling and ubiquitinates TLE1, TLE2, TLE3, TLE4, and AES. Ubiquitination of TLE3 results in inhibition of its interaction with TCF7L2/TCF4, thereby allowing efficient recruitment and binding of the transcriptional coactivator beta-catenin to TCF7L2/TCF4 that is required to initiate a Wnt-specific transcriptional program. |
| Complement factor H (CFH) | 0.781 | It is a glycoprotein that plays an essential role in maintaining a well-balanced immune response by modulating complement activation. Acts as a soluble inhibitor of complement, where its binding to self markers, such as glycan structures, prevents complement activation and amplification on cell surfaces. Accelerates the decay of the complement alternative pathway (AP) C3 convertase, C3bBb, thus preventing local formation of more C3b, the central player of the complement amplification loop. As a cofactor of the serine protease factor I, CFH also regulates proteolytic degradation of already-deposited C3b (PubMed: 18252712, PubMed: 28671664). In addition, mediates several cellular responses through interaction with specific receptors. For example, interacts with CR3/ITGAM receptor and thereby mediates the adhesion of human neutrophils to different pathogens. In turn, these pathogens are phagocytosed and destroyed. |
| Age-related maculopathy susceptibility 2 (ARMS2) | 0.781 | Age-related maculopathy susceptibility 2 |
| Microtubule-associated protein tau (MAPT) | 0.754 | MAPT promotes microtubule assembly and stability, and might be involved in the establishment and maintenance of neuronal polarity. The C-terminus binds axonal microtubules while the N-terminus binds neural plasma membrane components, suggesting that tau functions as a linker protein between both. Axonal polarity is predetermined by TAU/MAPT localization (in the neuronal cell) in the domain of the cell body defined by the centrosome. The short isoforms allow plasticity of the cytoskeleton whereas the longer isoforms may preferentially play a role in its stabilization. |
| ATP-dependent Clp protease proteolytic subunit (CLPP) | 0.742 | It is a protease component of the Clp complex that cleaves peptides and various proteins in an ATP-dependent process. Has low peptidase activity in the absence of CLPX. The Clp complex can degrade CSN1S1, CSN2, and CSN3, as well as synthetic peptides (*in vitro*) and may be responsible for a fairly general and central housekeeping function rather than for the degradation of specific substrates. Cleaves PINK1 in the mitochondrion. |
| Pleckstrin homology domain-containing family A member 1 (PLEKHA1) | 0.738 | PLEKHA1 binds specifically to phosphatidylinositol 3,4-diphosphate (PtdIns3,4P2), but not to other phosphoinositides. May recruit other proteins to the plasma membrane. |
| Macrophage migration inhibitory factor (MIF) | 0.674 | MIF is a pro-inflammatory cytokine involved in the innate immune response to bacterial pathogens. The expression of MIF at sites of inflammation suggests a role as mediator in regulating the function of macrophages in host defense. Counteracts the anti-inflammatory activity of glucocorticoids. Has phenylpyruvate tautomerase and dopachrome tautomerase activity (*in vitro*), but the physiological substrate is not known. It is not clear whether the tautomerase activity has any physiological relevance, and whether it is important for cytokine activity. |
| Beta-casein (CSN2) | 0.665 | It belongs to the beta-casein family, and plays an important role in determination of the surface properties of the casein micelles. |
| Chymotrypsin-C (CTRC) | 0.649 | It regulates activation and degradation of trypsinogens and pro-carboxypeptidases by targeting specific cleavage sites within their zymogen precursors. Has chymotrypsin-type protease activity and hypocalcemic activity. |

**Table 2c. Protein interaction partners of cathepsin G (CTSG), with associated functional annotations.** The interaction study was performed using STRING v12.0 tool. Screening was done against *Homo sapiens* as the organism of interest, following FDR stringency of 0.05, while adjusting the confidence score to 0.4.

| **Predicted functional partners of CTSG in STRING database** | **Interaction score** | **Annotated function according to literature and UniProt database** |
| --- | --- | --- |
| Myeloperoxidase heavy chain (MPO) | 0.999 | MPO belongs to peroxidase family, XPO subfamily. It is a part of host defense system of poly-morphonuclear (PMN) leukocytes, and is responsible for microbicidal activity against a wide range of organisms. In stimulated PMN, MPO catalyzes production of hypohalous acids, primarily hypochlorous acid in physiologic situations, and other toxic intermediates, greatly enhancing PMN microbicidal activity |
| Neutrophil elastase (ELANE) | 0.999 | It belongs to elastase subfamily, peptidase S1 family. Modifies the functions of natural killer cells, monocytes, and granulocytes. Inhibits C5a-dependent neutrophil enzyme release and chemotaxis. Capable of killing *Escherichia coli*, but not *Staphylococcus aureus in vitro*; digests outer membrane protein A (ompA) in *E. coli* and *Klebseilla pneumonia*. |
| Lactotransferrin (LTF) | 0.997 | Transferrins are iron binding transport proteins which can bind two Fe^3+^ ions in association with the binding of an anion, usually bicarbonate. Lactoferricin binds to the bacterial surface and is crucial for the bactericidal functions. Has some antiviral activity against papillomavirus infection. N-terminal region shows strong antifungal activity against *Candida albicans*. Contains two BBXB heparin-binding consensus sequences that appear to form the predominant functional GAG-binding site. Lactoferroxins A, B, and C have opioid antagonist activity. Lactoferroxin A shows preference for mu-receptors, while lactoferroxin B and C have somewhat higher degrees of preference for kappa-receptors than for mu-receptors. [Isoform DeltaLf]: transcription factor with antiproliferative properties and ability to induce cell cycle arrest. Binds to the DeltaLf response element found in the SKP1, BAX, DCPS, and SELENOH promoters. |
| Myeloblastin (PRTN3) | 0.997 | It belongs to the peptidase S1 family, elastase subfamily. It is a serine protease that degrades elastin, fibronectin, laminin, vitronectin, and collagen types I, III, and IV (*in vitro*). By cleaving and activating receptor F2RL1/PAR-2, enhances endothelial cell barrier function and thus vascular integrity during neutrophil transendothelial migration. May play a role in neutrophil transendothelial migration, probably when associated with CD177. |
| 67 kDa matrix metalloproteinase-9 (MMP9) | 0.992 | MMP9 belongs to the peptidase M10A family. May play an essential role in local proteolysis of the ECM and in leukocyte migration. Could play a role in bone osteoclastic resorption. Cleaves KiSS1 at a Gly-\|-Leu bond. Cleaves type IV and type V collagen into large C-terminal three quarter fragments and shorter N-terminal one quarter fragments. Degrades fibronectin but not laminin or Pz-peptide. |
| Alpha-1-antichymotrypsin His-Pro-less (SERPINA3) | 0.998 | Although its physiological function is unclear, it can inhibit neutrophil cathepsin G and mast cell chymase, both of which can convert angiotensin-1 to the active angiotensin-2. |
| Azurocidin (AZU1) | 0.977 | This is a neutrophil granule-derived antibacterial and monocyte- and fibroblast-specific chemotactic glycoprotein. Binds heparin. The cytotoxic action is limited to many species of Gram- negative bacteria; this specificity may be explained by a strong affinity of the very basic N-terminal half for the negatively charged lipopolysaccharides that are unique to the Gram-negative bacterial outer envelope. It may play a role in mediating recruitment of monocytes in the second wave of inflammation. Has antibacterial activity against the Gram-negative bacterium *P. aeruginosa*, this activity is inhibited by LPS from *P. aeruginosa*. Acting alone, it does not have antimicrobial activity against the Gram-negative bacteria *Aggregatibacter actinomycetemcomitans* ATCC 29532, *A. actinomycetemcomitans* NCTC 9709, *A.vactinomycetemcomitans* FDC-Y4, *Haemophilus aphrophilus* ATCC 13252, *Eikenella corrodens* ATCC 23834, *Capnocytophaga sputigena* ATCC 33123, *Capnocytophaga* spp. ATCC 33124, *Capnocytophaga* spp. ATCC 27872, or *E. coli* ML-35. Has antibacterial activity against *C. sputigena* ATCC 33123 when acting synergistically with either elastase or cathepsin G. |
| Serpin B4 (SERPINB4) | 0.973 | It may act as a protease inhibitor to modulate the host immune response against tumor cells. |
| Cathelicidin antimicrobial peptide (CAMP) | 0.971 | It binds to bacterial lipopolysaccharides (LPS), and has antibacterial activity. |
| Bactericidal permeability-increasing protein (BPI) | 0.959 | BPI belongs to the BPI/LBP/Plunc superfamily, BPI/LBP family. The cytotoxic action of BPI is limited to many species of Gram-negative bacteria; this specificity may be explained by a strong affinity of the very basic N-terminal half for the negatively charged lipopolysaccharides that are unique to the Gram-negative bacterial outer envelope. Has antibacterial activity against the Gram-negative bacterium *P. aeruginosa*, this activity is inhibited by LPS from *P. aeruginosa*. |

**Table 2d. Protein interaction partners of alpha-1-acid glycoprotein 1 (AGP1), with associated functional annotations.** The interaction study was performed using STRING v12.0 tool. Screening was done against *Homo sapiens* as the organism of interest, following FDR stringency of 0.05, while adjusting the confidence score to 0.4.

| **Predicted functional partners of AGP1 in STRING database** | **Interaction score** | **Annotated function according to literature and UniProt database** |
| --- | --- | --- |
| Alpha-1-acid glycoprotein 2 (ORM2) | 0.999 | Functions as transport protein in the blood stream. Binds various hydrophobic ligands in the interior of its beta-barrel domain. Also binds synthetic drugs and influences their distribution and availability. Appears to function in modulating the activity of the immune system during the acute-phase reaction. |
| Serum albumin (ALB) | 0.998 | It is the main protein of plasma, has a good binding capacity for water, Ca^2+^, Na^+^, K^+^, fatty acids, hormones, bilirubin and drugs (probable). Its main function is the regulation of the colloidal osmotic pressure of blood (probable). Major zinc transporter in plasma, typically binds about 80% of all plasma zinc. Major calcium and magnesium transporter in plasma, binds approximately 45% of circulating calcium and magnesium in plasma (by similarity). Potentially has more than two calcium-binding sites and might additionally bind calcium in a non-specific manner (by similarity). The shared binding site between zinc and calcium at residue Asp-273 suggests a crosstalk between zinc and calcium transport in the blood (by similarity). The rank order of affinity is zinc > calcium > magnesium (by similarity). Binds to the bacterial siderophore enterobactin and inhibits enterobactin-mediated iron uptake of *E. coli* from ferric transferrin, and may thereby limit the utilization of iron and growth of enteric bacteria such as *E.coli*. Does not prevent iron uptake by the bacterial siderophore aerobactin. |
| Short peptide from AAT (SERPINA1) | 0.979 | SERPINA1 belongs to the serpin family. It is inhibitor of serine proteases. Its primary target is elastase, but it also has a moderate affinity for plasmin and thrombin. Irreversibly inhibits trypsin, chymotrypsin, and plasminogen activator. The aberrant form inhibits insulin-induced NO synthesis in platelets, decreases coagulation time and has proteolytic activity against insulin and plasmin. |
| Haptoglobin alpha chain (HP) | 0.973 | As a result of hemolysis, hemoglobin is found to accumulate in the kidney and is secreted in the urine. Haptoglobin captures, and combines with free plasma hemoglobin to allow hepatic recycling of heme iron and to prevent kidney damage. Haptoglobin also acts as an antioxidant, has antibacterial activity, and plays a role in modulating many aspects of the acute phase response. Hemoglobin/ haptoglobin complexes are rapidly cleared by the macrophage CD163 scavenger receptor expressed on the surface of liver Kupfer cells through an endocytic lysosomal degradation pathway. |
| Serine palmitoyltransferase 1 (SPTLC1) | 0.943 | The heterodimer formed with SPTLC2 or SPTLC3 constitutes the catalytic core. Composition of the SPT complex determines the substrate preference. The SPTLC1-SPTLC2-SPTSSA complex shows a strong preference for C16-CoA substrate, while the SPTLC1-SPTLC3-SPTSSA isozyme uses both C14-CoA and C16-CoA as substrates, with a slight preference for C14-CoA. The SPTLC1-SPTLC2-SPTSSB complex shows a strong preference for C18-CoA substrate, while the SPTLC1-SPTLC3-SPTSSB isozyme displays an ability to use a broader range of acyl-CoAs, without apparent preference. Required for adipocyte cell viability and metabolic homeostasis (by similarity). |
| Alpha-2-HS-glycoprotein chain A (AHSG) | 0.926 | It belongs to the fetuin family. Promotes endocytosis, possesses opsonic properties, and influences the mineral phase of bone. Shows affinity for calcium and barium ions. |
| Ceruloplasmin (CP) | 0.923 | CP is a blue, copper-binding (6-7 atoms per molecule) glycoprotein. It has ferroxidase activity oxidizing Fe^2+^ to Fe^3+^ without releasing radical oxygen species. Involved in iron transport across cell membrane. Provides Cu^2+^ ions for the ascorbate-mediated deaminase degradation of the heparan sulfate chains of GPC1. May also play a role in fetal lung development or pulmonary antioxidant defense (by similarity). |
| Inter-alpha-trypsin inhibitor light chain (AMBP) | 0.922 | It belongs to the calycin superfamily, lipocalin family. Inter-alpha-trypsin inhibitor inhibits trypsin, plasmin, and lysosomal granulocytic elastase. Inhibits calcium oxalate crystallization. |
| Alpha-1-antichymotrypsin His-Pro-less (SERPINA3) | 0.918 | Although its physiological function is unclear, it can inhibit neutrophil cathepsin G and mast cell chymase, both of which can convert angiotensin-1 to the active angiotensin-2. |
| Serine palmitoyltransferase 3 (SPTLC3) | 0.912 | The heterodimer formed with LCB1/SPTLC1 constitutes the catalytic core. Composition of SPT complex determines substrate preference. SPT complexes containing SPTLC3 generate shorter chain sphingoid bases compared to complexes containing SPTLC2. SPTLC1- SPTLC3-SPTSSA isozyme uses C12-CoA, C14-CoA and C16-CoA as substrates, with a slight preference for C14-CoA. On the other hand, SPTLC1-SPTLC3-SPTSSB has the ability to use a broader range of acyl-CoAs without apparent preference. |

**Table 3. Biological significance of the pathways identified from Reactome database-based functional enrichment analysis of various interactomes generated using STRING v12.0, as deduced from the literature.**

| **Protein(s) utilized for interactome generation using STRING v12.0 tool** | **Pathways identified from Reactome database-based functional enrichment analysis of the interactome** | **Biological relevance of the STRING interaction network in OADM, based on published literature** |
| --- | --- | --- |
| All the significant differentially abundant key glycated proteins in OADM disease condition [Interactome described in Fig. 7A and Additional Table 2a] | Common pathway of fibrin clot formation, plasma lipoprotein remodeling, regulation of insulin-like growth factor (IGF) transport and uptake by IGF-binding proteins (IGFBPs), post-translational protein phosphorylation, platelet degranulation, chylomicron remodeling, and chylomicron assembly [Fig. 7B] | Enrichment of pathways related to fibrin clot formation and plasma lipoprotein remodeling suggests a potential contribution of vascular dysfunction and lipid dysregulation to cartilage degeneration in OA, as reported previously (Hügle et al., 2022^[[1]](#footnote-2)^; Adam et al., 2024^[[2]](#footnote-3)^). The enrichment of pathways involved in insulin-like growth factor (IGF) transport and uptake by IGF-binding proteins (IGFBPs), as well as post-translational protein phosphorylation, is indicative of altered metabolic regulation, processes that are critical for maintaining cartilage homeostasis and systemic glucose balance (Baxter, 2023^[[3]](#footnote-4)^; Zhong et al., 2023^[[4]](#footnote-5)^). In addition, enrichment of neutrophil degranulation and chylomicron assembly and remodeling pathways highlights the involvement of inflammatory responses and lipid metabolic disturbances that may contribute to tissue damage and associated metabolic complications (Xiao et al., 2019^[[5]](#footnote-6)^; Zhang et al., 2024)^[[6]](#footnote-7)^, in OADM. Collectively, these findings support the concept that structural remodeling, inflammation, and metabolic dysregulation are interconnected processes in OADM, and suggest that components of these pathways may warrant further investigation as potential targets for therapeutic intervention. |
| High-temperature requirement protein A1 (HTRA1) [Interactome described in Fig. 7C and Additional Table 2b] | Regulation of complement cascade, and activation of C3 and C5 complement proteins [Fig. 7D] | Reactome pathway enrichment related to regulation of the complement cascade highlights the potential involvement of immune activation and inflammatory processes in OADM. In particular, complement activation components, including C3 and C5, have been implicated in joint inflammation in OA and have also been associated with systemic inflammatory states linked to insulin resistance in T2DM (Assirelli et al., 2020^[[7]](#footnote-8)^; Shim et al., 2020^[[8]](#footnote-9)^). These observations suggest that dysregulated complement signaling may represent a shared inflammatory axis contributing to both local joint pathology and metabolic dysfunction in OADM. |
| Cathepsin G (CTSG) [Interactome described in Fig. 7E and Additional Table 2c] | Neutrophil degranulation, the activity of antimicrobial peptides, and the activation of matrix metalloproteinases [Fig. 7F] | The enrichment of Reactome pathways such as neutrophil degranulation, which releases proteases like the ECM-degrading CTSG protease, and the activation of MMPs, is consistent with a potential role of immune and inflammatory processes in contributing to cartilage degradation and metabolic alterations (Mukherjee and Das, 2024^[[9]](#footnote-10)^), observed in OADM. |
| Alpha-1-acid glycoprotein 1 (AGP1) [Interactome described in Fig. 7G and Additional Table 2d] | Platelet and neutrophil degranulation, scavenging of heme from plasma, post-translational protein phosphorylation, and regulation of IGF transport and uptake by IGFBPs [Fig. 7H] | The Reactome pathways analysis associated AGP1 with pathways related to platelet and neutrophil degranulation, scavenging of heme, post-translational protein phosphorylation, regulation of IGF transport and uptake by IGFBPs, which are potentially linked to inflammation and tissue remodeling in disease conditions, including OA and T2DM (Levy et al., 2007^[[10]](#footnote-11)^; Zhang et al., 2024^[[11]](#footnote-12)^). |

**ADDITIONAL FIGURE CAPTIONS**

1. **Fig. 1. Age, body mass index (BMI), fasting blood sugar (FBS), and glycated hemoglobin (HbA1c) of patients, enrolled in different study groups, and considered for (a) discovery and verification, and (b) validation phase analysis, along with total protein concentration (TPC) of patient synovial fluid and serum samples.**

**Fig. 1a. (A) Age, (B) body mass index (BMI), (C) fasting blood sugar (FBS), and (D) glycated hemoglobin (HbA1c) of patients enrolled in different study groups, and considered for discovery and verification phase analysis, along with total protein concentration (TPC) of patient (E) synovial fluid and (F) serum samples.** Data normality was formally assessed using both Shapiro-Wilk and Lilliefors-corrected Kolmogorov-Smirnov tests. Normal data, herein, age, FBS, and TPC, were subjected to parametric Welch’s *t*-test for significance, and are presented as Mean ± SD. Skewed data, herein, BMI, and HbA1c, were subjected to non-parametric Mann-Whitney test for significance, and are presented as Median (IQR). **p* < 0.05, and ***p* < 0.01 represent statistically significant data, while ns indicates non-significant data.


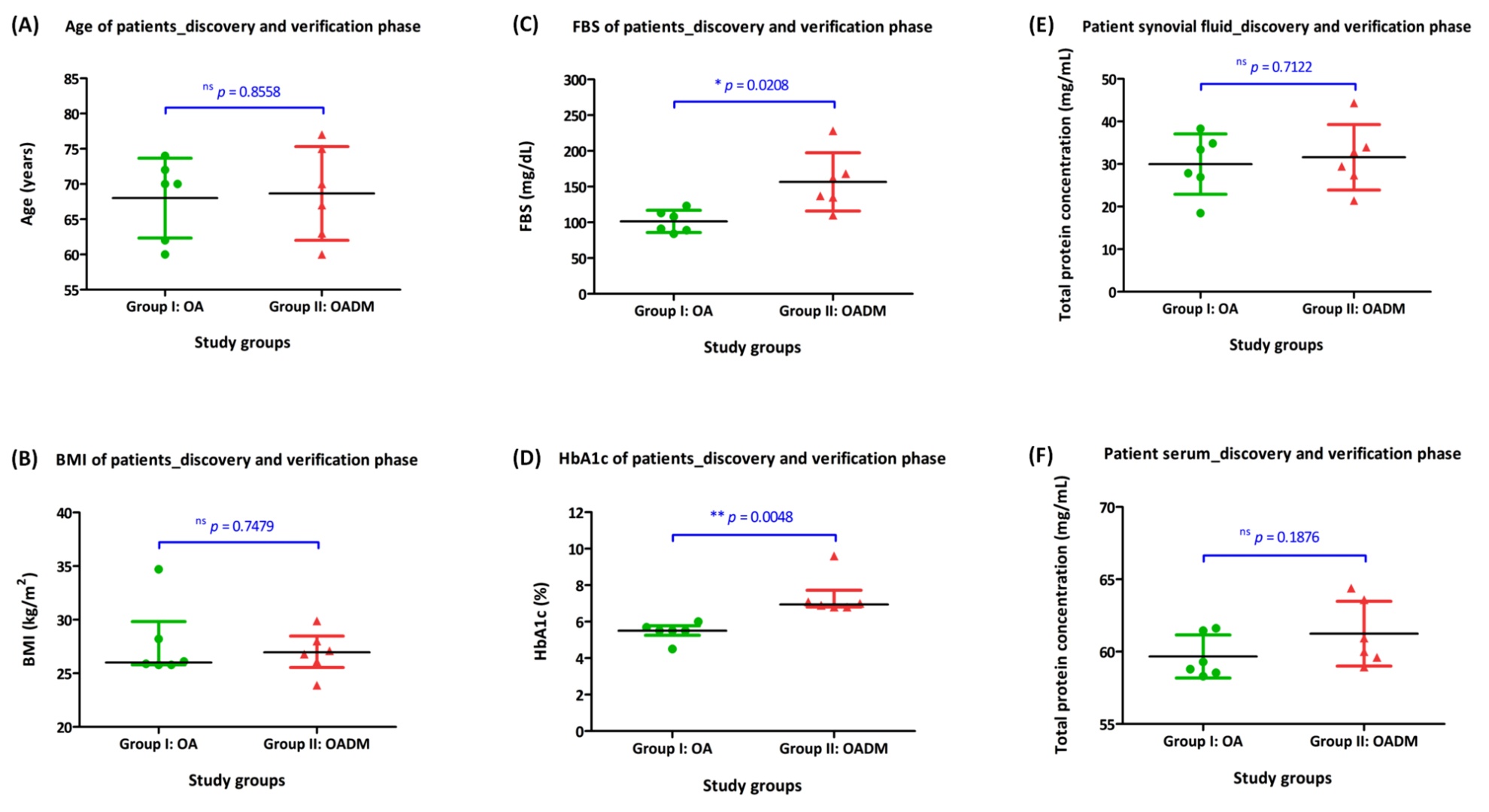


**Fig. 1b. (A) Age, (B) body mass index (BMI), (C) fasting blood sugar (FBS), and (D) glycated hemoglobin (HbA1c) of patients enrolled in different study groups, and considered for validation phase analysis, along with total protein concentration (TPC) of patient (E) synovial fluid and (F) serum samples.** Data normality was formally assessed using both Shapiro-Wilk and Lilliefors-corrected Kolmogorov-Smirnov tests. Normal data, herein, age, FBS, and TPC, were subjected to parametric Welch’s *t*-test for significance, and are presented as Mean ± SD. Skewed data, herein, BMI, and HbA1c, were subjected to non-parametric Mann-Whitney test for significance, and are presented as Median (IQR). *****p* < 0.0001 represents statistically significant data, while ns indicates non-significant data.


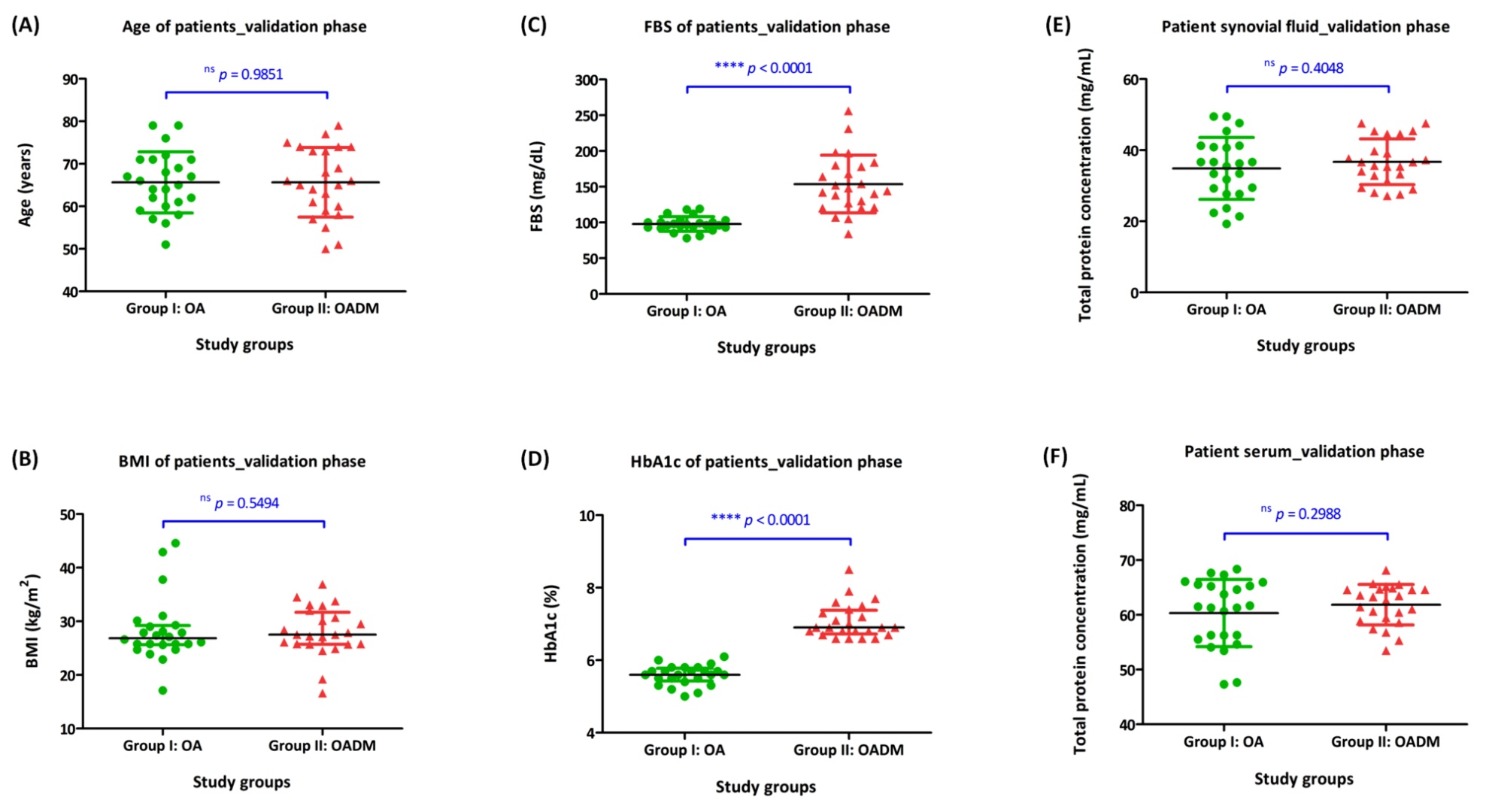


1. Hügle T, Nasi S, Ehirchiou D, Omoumi P, So A, Busso N. Fibrin deposition associates with cartilage degeneration in arthritis. EBioMedicine. 2022 Jul 1;81. [↑](#footnote-ref-2)
2. Adam MS, Zhuang H, Ren X, Zhang Y, Zhou P. The metabolic characteristics and changes of chondrocytes in vivo and in vitro in osteoarthritis. Frontiers in endocrinology. 2024 May 24;15:1393550. [↑](#footnote-ref-3)
3. Baxter RC. Signaling pathways of the insulin-like growth factor binding proteins. Endocrine reviews. 2023 Sep 15;44(5):753-78. [↑](#footnote-ref-4)
4. Zhong Q, Xiao X, Qiu Y, Xu Z, Chen C, Chong B, Zhao X, Hai S, Li S, An Z, Dai L. Protein posttranslational modifications in health and diseases: Functions, regulatory mechanisms, and therapeutic implications. MedComm. 2023 Jun;4(3):e261. [↑](#footnote-ref-5)
5. Xiao C, Stahel P, Lewis GF. Regulation of chylomicron secretion: focus on post-assembly mechanisms. Cellular and molecular gastroenterology and hepatology. 2019 Jan 1;7(3):487-501. [↑](#footnote-ref-6)
6. Zhang F, Xia Y, Su J, Quan F, Zhou H, Li Q, Feng Q, Lin C, Wang D, Jiang Z. Neutrophil diversity and function in health and disease. Signal transduction and targeted therapy. 2024 Dec 6;9(1):343. [↑](#footnote-ref-7)
7. Assirelli E, Pulsatelli L, Dolzani P, Mariani E, Lisignoli G, Addimanda O, Meliconi R. Complement expression and activation in osteoarthritis joint compartments. Frontiers in Immunology. 2020 Oct 29;11:535010. [↑](#footnote-ref-8)
8. Shim K, Begum R, Yang C, Wang H. Complement activation in obesity, insulin resistance, and type 2 diabetes mellitus. World journal of diabetes. 2020 Jan 15;11(1):1. [↑](#footnote-ref-9)
9. Mukherjee A, Das B. The role of inflammatory mediators and matrix metalloproteinases (MMPs) in the progression of osteoarthritis. Biomaterials and biosystems. 2024 Mar 1;13:100090. [↑](#footnote-ref-10)
10. Levy AP, Purushothaman KR, Levy NS, Purushothaman M, Strauss M, Asleh R, Marsh S, Cohen O, Moestrup SK, Moller HJ, Zias EA. Downregulation of the hemoglobin scavenger receptor in individuals with diabetes and the Hp 2-2 genotype: implications for the response to intraplaque hemorrhage and plaque vulnerability. Circulation research. 2007 Jul 6;101(1):106-10. [↑](#footnote-ref-11)
11. Zhang F, Xia Y, Su J, Quan F, Zhou H, Li Q, Feng Q, Lin C, Wang D, Jiang Z. Neutrophil diversity and function in health and disease. Signal transduction and targeted therapy. 2024 Dec 6;9(1):343. [↑](#footnote-ref-12)
